# The genome sequence of *Streptomyces rochei* 7434AN4, which carries a linear chromosome and three characteristic linear plasmids

**DOI:** 10.1038/s41598-019-47406-y

**Published:** 2019-07-29

**Authors:** Yosi Nindita, Zhisheng Cao, Amirudin Akhmad Fauzi, Aiko Teshima, Yuya Misaki, Rukman Muslimin, Yingjie Yang, Yuh Shiwa, Hirofumi Yoshikawa, Michihira Tagami, Alexander Lezhava, Jun Ishikawa, Makoto Kuroda, Tsuyoshi Sekizuka, Kuninobu Inada, Haruyasu Kinashi, Kenji Arakawa

**Affiliations:** 1 Department of Molecular Biotechnology, Graduate School of Advanced Sciences of Matter, Hiroshima University, 1-3-1 Kagamiyama, Higashi-Hiroshima, 739-8530 Japan; 2 Unit of Biotechnology, Division of Biological and Life Sciences, Graduate School of Integrated Sciences for Life, Hiroshima University, 1-3-1 Kagamiyama, Higashi-Hiroshima, 739-8530 Japan; 3 NODAI Genome Research Center, Tokyo University of Agriculture, 1-1-1 Sakuragaoka, Setagaya-ku, Tokyo 156-8502 Japan; 4 Department of Bioscience, Tokyo University of Agriculture, 1-1-1 Sakuragaoka, Setagaya-ku, Tokyo 156-8502 Japan; 5 Omics Science Center, RIKEN, 1-7-22 Suehiro-cho, Tsurumi-ku, Yokohama, Kanagawa 230-0045 Japan; 6 Department of Bioactive Molecules, National Institute of Infectious Diseases, 1-23-1 Toyama, Shinjuku-ku, Tokyo 162-8640 Japan; 7 Pathogen Genomics Center, National Institute of Infectious Diseases, 1-23-1 Toyama, Shinjuku-ku, Tokyo 162-8640 Japan; 8 Natural Science Center for Basic Research and Development, Hiroshima University, 1-4-2 Kagamiyama, Higashi-Hiroshima, 739-8526 Japan

**Keywords:** Genome, Biosynthesis

## Abstract

*Streptomyces rochei* 7434AN4 produces two structurally unrelated polyketide antibiotics, lankacidin and lankamycin, and carries three linear plasmids, pSLA2-L (211 kb), -M (113 kb), and -S (18 kb), whose nucleotide sequences were previously reported. The complete nucleotide sequence of the *S. rochei* chromosome has now been determined using the long-read PacBio RS-II sequencing together with short-read Illumina Genome Analyzer IIx sequencing and Roche 454 pyrosequencing techniques. The assembled sequence revealed an 8,364,802-bp linear chromosome with a high G + C content of 71.7% and 7,568 protein-coding ORFs. Thus, the gross genome size of *S. rochei* 7434AN4 was confirmed to be 8,706,406 bp including the three linear plasmids. Consistent with our previous study, a *tap-tpg* gene pair, which is essential for the maintenance of a linear topology of *Streptomyces* genomes, was not found on the chromosome. Remarkably, the *S. rochei* chromosome contains seven ribosomal RNA (*rrn*) operons (16S-23S-5S), although *Streptomyces* species generally contain six *rrn* operons. Based on 2ndFind and antiSMASH platforms, the *S. rochei* chromosome harbors at least 35 secondary metabolite biosynthetic gene clusters, including those for the 28-membered polyene macrolide pentamycin and the azoxyalkene compound KA57-A.

## Introduction

The filamentous Gram-positive soil bacterial genus *Streptomyces* is well characterized by its prolific potential to produce a vast array of secondary metabolites, including agriculturally and clinically useful antibiotics. Unlike other bacteria, their chromosomes are linear with a size of around 8–9 Mb. *Streptomyces* linear replicons harbor terminal inverted repeat (TIR) sequences at both ends and the 5′-ends are covalently bound to terminal protein (TP)^1^. *Streptomyces* linear chromosomes frequently undergo spontaneous deletions, leading to DNA rearrangements including amplification, arm replacement, and circularization^2–4^.

To date, over 1,141 *Streptomyces* strains have been sequenced and deposited in the GenBank database (ftp://ftp.ncbi.nlm.nih.gov/genomes/genbank/bacteria/) (as of 26th of June 2019), including the actinorhodin producer *Streptomyces coelicolor* A3(2)^5^, the avermectin producer *Streptomyces avermitilis*^6^, and the streptomycin producer *Streptomyces griseus*^7^ (Table 1). *Streptomyces* species have a great potential to produce over 20 secondary metabolites, including polyketides, non-ribosomal peptides, terpenoids, aminoglycosides, siderophores, and others. However, most of these biosynthetic gene clusters are poorly expressed or are not at all under normal culture conditions. Thus, *Streptomyces* genomes are a valuable source for natural product discovery.

**Table 1:** General features of the chromosomes of *Streptomyces rochei* 7434AN4 and five other *Streptomyces* species.

Species^a^	Length (bp)	G + C content (%)	CDS (no.)	Average CDS length (bp)	TIR (bp)	rRNAs (no.)	tRNA (no.)
*S. rochei* 7434AN4	8,364,802	71.7	7,568	973	53,892	7	67
*S. coelicolor* A3(2)	8,667,507	72.1	7,825	991	21,653	6	63
*S. avermitilis* MA-4680	9,025,608	70.7	7,582	1,027	49	6	68
*S. griseus* IFO13350	8,545,929	72.2	7,138	1,055	132,910	6	66
*S. hygroscopicus* 5008	10,145,833	71.9	8,849	952	14	6	68
*S. scabies* 87.22	10,148,695	71.5	8,746	1,005	18,488	6	75

^a^GenBank accession number: *S. rochei* 7434AN4 chromosome, AP018517; *S. coelicolor* A3(2), NC_003888; *S. avermitilis* MA-4680, NC_003155; *S. griseus* IFO13350, NC_010572; *S. hygroscopicus* 5008, CP003275; *S. scabies* 87.22, NC_013929.

**Figure 1:**
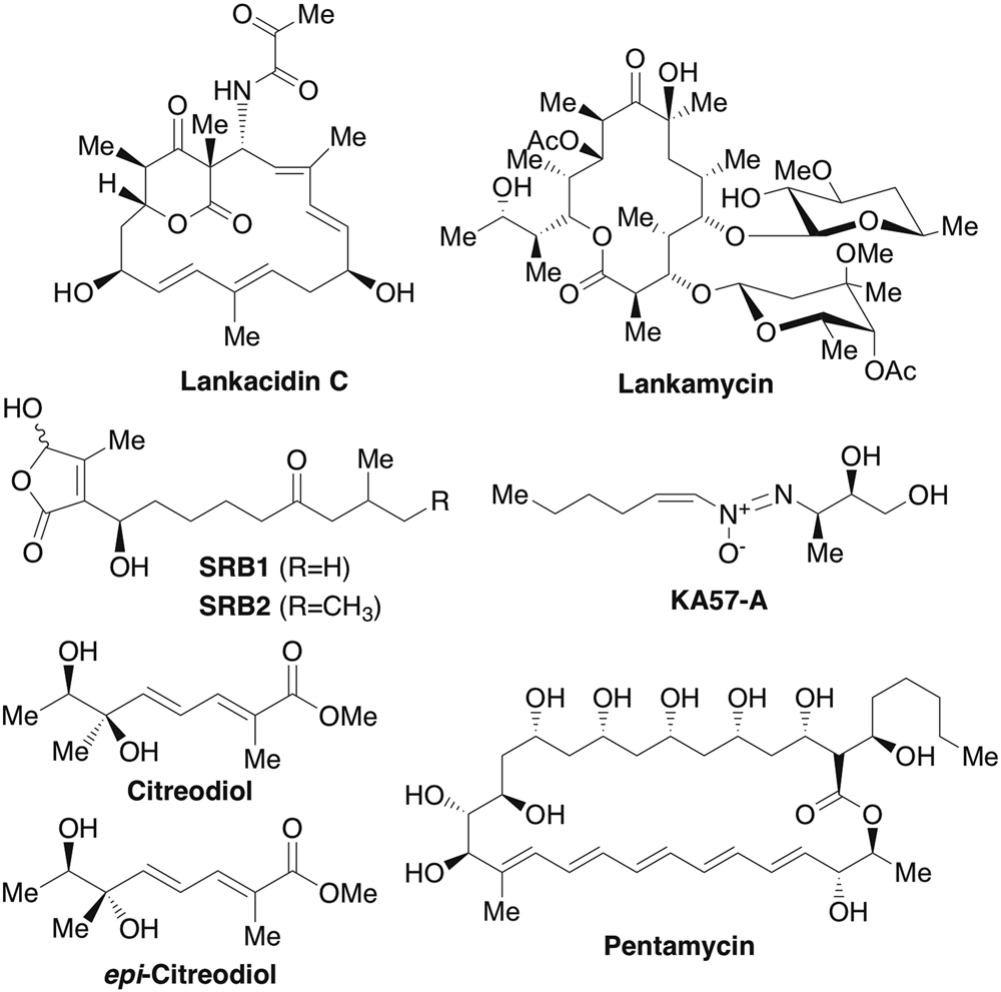
Secondary metabolites produced by *Streptomyce rochei* 7434AN4 and its mutants. Polyketide antibiotics; lankacidin C and lankamycin. Signaling molecules; SRB1 and SRB2. Azoxyalkene; KA57-A. Polyketides; citreodiol, *epi*-citreodiol, and pentamycin.

*Streptomyces rochei* 7434AN4 produces two structurally unrelated polyketides, lankacidin C and lankamycin (Fig. 1)^8,9^, and carries three characteristic linear plasmids (pSLA2-L, -M, and -S), whose nucleotide sequences were previously determined (Table 2). The 210,614-bp largest linear plasmid, pSLA2-L (Accession number; AB088224), carries 143 open reading frames (ORFs), including the biosynthetic gene clusters for lankacidin, lankamycin, uncharacterized type-II polyketide, and carotenoid^10^. This plasmid carries many regulatory genes and the biosynthetic gene for the signaling molecules SRBs (*Streptomyces rochei* butenolides) (Fig. 1) that induce lankacidin and lankamycin production in *S. rochei*^11^. The 113,464-bp linear plasmid pSLA2-M (AB597522) comprises of 121 ORFs and carries several self-defense genes including a CRISPR (clustered regularly interspaced short palindromic repeats) cluster and a *ku70/ku80*-like gene^12^. Both plasmids harbor a *tap*-*tpg* gene pair that encodes a telomere-associated protein and a TP necessary for end patching of linear replicons^13^. The 17,526-bp smallest plasmid pSLA2-S (AB905437) consists of 17 ORFs and does not contain a *tap-tpg* gene pair in contrast to pSLA2-L and -M. Basic features of the three linear plasmids are shown in Table 2.

**Table 2:** General features of the chromosome and three plasmids of *S. rochei* 7434AN4.

Replicons^a^	Length (bp)	G + C content (%)	CDS (no.)	Average CDS length (bp)	TIR (bp)
Chromosome	8,364,802	71.7	7,568	973	53,892
pSLA2-L	210,614	72.8	143	1,330	1,992
pSLA2-M	113,464	69.7	121	718	352
pSLA2-S	17,526	69.7	22	508	817

^a^GenBank accession number: *S. rochei* 7434AN4 chromosome, AP018517; pSLA2-L, AB088224; pSLA2-M, AB597522; pSLA2-S, AB905437.

Regarding the genomic structures in *S. rochei* 7434AN4, the right end sequences of pSLA2-L and -M are almost identical (99.9%) up to 14.6 kb from the end^12^. Partial sequencing and Southern blot analysis revealed that both ends of the chromosome are identical to each other, and share 98.5% homology with the right end of pSLA2-L and -M up to 3.1 kb. Furthermore, a truncated *tpg* homolog was detected in one contig^14^. In addition, curing of pSLA2-L from strain 51252, which harbors only pSLA2-L, caused terminal deletions of the chromosome followed by circularization in mutant 2–39^9,14^. These results suggest that the *tap-tpg* of pSLA2-L or -M functions for terminal replication to maintain a linear topology of the chromosome. This hypothesis was supported by complementation and curing experiments of the *tap*-*tpg* of pSLA2-M^14^. However, the absence of a *tap-tpg* pair on the chromosome still remained to be proved by genome sequencing.

*Streptomyces* species are well known for their three characteristics: possession of linear replicons, complex morphological differentiation, and an ability to produce secondary metabolites. In this study, we have determined the complete nucleotide sequence of the linear chromosome of *Streptomyces rochei* 7434AN4 and extensively analyzed these three characteristics in comparison with other *Streptomyces* strains hitherto characterized.

## Results and Discussion

### Nucleotide sequencing and physical analysis of the linear chromosome of *S. rochei* 7434AN4

**Figure 2:**
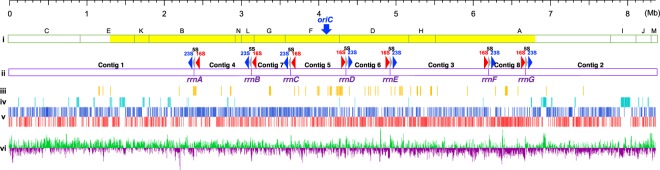
Schematic representation of the *S. rochei* chromosome. Scale bars are drawn in megabases. (**i**) *Ase*I physical map. The possible core region of the *S. rochei* chromosome (1.31–6.80 Mb) is marked as yellow. (**ii**) Distribution of rRNA-encoding gene (rDNA) operons and eight assembled contigs. Gray and blue arrows are 5S-23S rDNA operon, while red is 16S rDNA. (**iii**) Distribution of tRNAs. (**iv**) Distribution of secondary metabolite gene clusters. (**v**) Distribution of CDSs according to direction of transcription (+ strand, upper line; − strand, lower line). (**vi**) GC-skew for 10-kb window and 500-bp step. The putative *oriC* (gene) locus is indicated by a blue arrow. Main features were generated by DNA plotter software (https://www.sanger.ac.uk/science/tools/dnaplotter)^15^.

The complete nucleotide sequence of the linear chromosome of *S. rochei* 7434AN4 was obtained by assembling a combination of reads from the long-read PacBio RS-II sequencing together with short-read Illumina Genome Analyzer IIx (GAIIx) sequencing and Roche 454 pyrosequencing. The 26,119,215 trimmed reads (217-fold coverage of the whole genome) obtained through Illumina GAIIx sequencing were assembled using ABySS protocol to give 340 contigs with >500 bp length. Then, the PacBio RS-II sequencing independently generated 598.1 Mb of sequence data (69-fold coverage). After extensive read assembly and correction among these sequencings with the help of sequence data from Roche 454 pyrosequencing, eight contigs were obtained (Fig. 2). Contigs 1 and 2 harbored a 3.1-kb homologous sequence with the right end of pSLA2-L and -M, locating them at both ends of the chromosome. Opposite boundaries in contigs 1 and 2 harbored downstream of 5S-23S rRNA-encoding genes (rDNAs) (gray and blue arrows, respectively, in Fig. 2-ii). Five contigs, 3, 4, 6, 7, and 8, contained downstream of 5S-23S rDNAs and upstream of 16S rDNA (red arrows in Fig. 2-ii) at both ends. Contig 5 harbored 16S rDNA at both ends, indicating the presence of seven rRNA operons (16S-23S-5S) on the *S. rochei* chromosome. Unlinked contig gaps were filled by conventional PCR amplification using KOD-plus Neo DNA polymerase and some group-specific 16S and 5S rRNA primer sets (Table S1). Seven amplified PCR fragments (ca. 6 kb) covering each rRNA operon were sequenced and the connectivities of eight contigs were confirmed. The final assembled sequence revealed an 8,364,802-bp linear chromosome with a G + C content of 71.7% and 7,568 predicted coding DNA sequences (CDSs). Since we previously reported the nucleotide sequences of the three linear plasmids, pSLA2-L (210,614 bp)^10^, -M (113,464 bp)^12^, and -S (17,526 bp)^14^, the gross genomic sequence of strain 7434AN4 has now been determined to be 8,706,406 bp.

The linear topology of the chromosome was further supported by pulsed-field gel electrophoresis (PFGE) analysis of *Ase*I and *Dra*I digest (Fig. S2). All of the predicted *Ase*I fragments, namely six fragments larger than 610 kb, three fragments ranging 285–450 kb, and five fragments below 225 kb were detected, except for the largest fragment *Ase*I-A (~2.25 Mb). Southern hybridization analysis of PFGE fragments probed by PCR fragments containing a flanking *Ase*I site agreed with the calculated *Ase*I map (Fig. S2). All of the predicted *Dra*I fragments, four larger than 945 kb, three ranging 285–450 kb, and three below 225 kb, were detected (Fig. S2).

### General features of the linear chromosome of *S. rochei* 7434AN4

**Figure 3:**
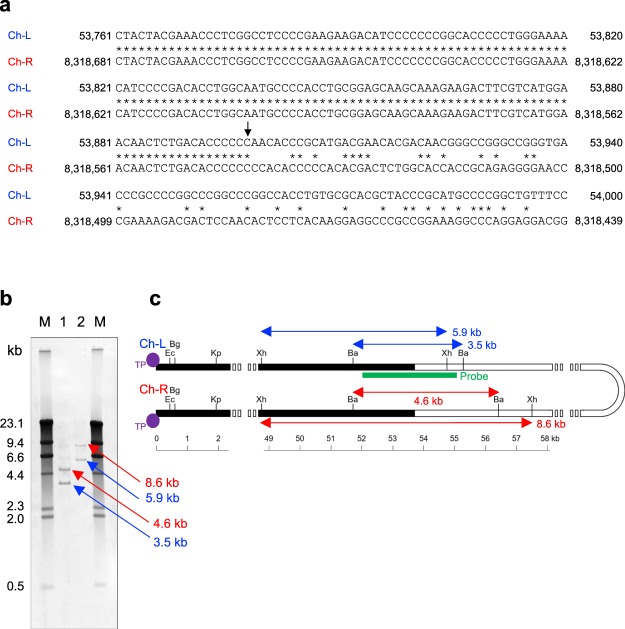
Terminal inverted repeat (TIR) of the *S. rochei* chromosome. (**a**) Nucleotide sequence comparison of inside ends of the TIR regions. Identical sequences are indicated by asterisks. Ch-L, chromosome left region; Ch-R, chromosome right region. (**b**) Southern hybridization analysis of the TIR regions. PCR fragment harboring nt 52,079–55,053 was used as a DNA probe to distinguish the TIR boundary. λ-DNA digested with *Hin*dIII was used as a DNA size marker. Lane M, λ/*Hin*dIII marker; lane 1, *S. rochei* 7434AN4 total DNA digested with *Bam*HI; lane 2, *S. rochei* 7434AN4 total DNA digested with *Xho*I. (**c**) Restriction map of the left and right TIR regions of the *S. rochei* 7434AN4 chromosome. TIR regions of the chromosome are shown by thick black lines. Terminal proteins attached to the 5′-ends are indicated by filled circles. Some important restriction sites are indicated. Bg, *Bgl*II; Ba, *Bam*HI; Xh, *Xho*I; Ec, *Eco*RI; Kp, *Kpn*I.

The general features of the *S. rochei* 7434AN4 chromosome are summarized in Tables 1 and 2. In addition, features including distribution of rDNA operons, tRNAs, BGCs, and CDSs according to direction of transcription (+ strand, upper line; − strand, lower line) as well as GC-skew diagram (Fig. 2) were generated by DNA plotter software (https://www.sanger.ac.uk/science/tools/dnaplotter)^15^. Linear chromosomes and linear plasmids of *Streptomyces* generally contain TIRs at both ends^1^. Previous Southern blot analysis with the pSLA2-L end probe indicated that the size of the TIRs of the 7434AN4 chromosome is shorter than 70 kb^14^. The present study revealed that the 7434AN4 chromosome has 53,892-bp TIRs (Fig. 3a). The inside ends of the TIRs were analyzed by Southern hybridization using a 3.0-kb PCR fragment (nt 52,079–55,053) as a probe (Fig. 3b). When 7434AN4 total DNA was digested with *Bam*HI, two expected signals appeared at 3.5 kb (left-region of chromosome = ch-L) and 4.6 kb (right-region of chromosome = ch-R). When digested with *Xho*I, two signals were observed at 5.9 kb (ch-L) and 8.6 kb (ch-R). These results coincide with the obtained sequence data. The length of TIR varies among *Streptomyces* species, generally from tens to hundreds kilobases^16^. Ubiquitous presence of relatively long TIRs at both ends of *Streptomyces* linear replicons led to the idea that they might function to maintain a linear topology^17^. TIRs potentially provide a suitable location for homologous recombination, when one TIR is lost by terminal deletion. If recombination occurs inside of TIRs, it regenerates intact TIRs and if does outside of TIRs, it results in arm replacement^18,19^, both of which could recover intact termini. However, exceptionally short TIRs were found in *S. hygroscopicus* 5008 (14 bp)^20^ and in *S. avermitilis* (49 bp)^6^ (Table 1).

Telomere-associated protein and terminal protein coded by *tap* and *tpg*, respectively, are necessary for terminal replication of *Streptomyces* linear replicons^13^. However, only a truncated *tpg* homolog (SRO_0241, 136 aa) without a counterpart of *tap* was found on the *S. rochei* chromosome. This gene is identical to the truncated *tpg* homolog in contig 95 reported previously by our group^14^ and shows a short but high homology only to the central part of *tpg*; the gene product of SRO_0241 (136 aa) shows 84% (29/35) and 69% (24/35) identity to that of *tpgR1* of pSLA2-L (184 aa) and *tpgRM* of pSLA2-M (184 aa), respectively. This result suggests that the original *tpg* gene on the chromosome might have been truncated at both 5′- and 3′-sides to generate a fusion gene SRO_0241 with no function. Thus, the absence of a *tap-tpg* gene pair on the chromosome of strain 7434AN4 has now been confirmed by genome sequencing. In addition, our hypothesis that the lack of *tap-tpg* is rescued in *S. rochei* 7434AN4 by introducing pSLA2-L and -M^14^, has also been proved. In this connection, the smallest plasmid pSLA2-S (17,526 bp) lacks a *tap*-*tpg* gene pair. It is reasonable that we could not obtain a mutant carrying only pSLA2-S from *S. rochei* 7434AN4, although all other mutants carrying possible combinations of three linear plasmids were obtained^9^.

The *S. rochei* chromosome contained seven rRNA operons (Fig. 2 and Table S2) and 67 tRNA genes (from 43 families) (Table S3). The replication origin *oriC* of *Streptomyces* is generally located between *dnaA* and *dnaN*, and contains at least 19 *dnaA* box-like sequences^21^. A putative *oriC* of the *S. rochei* 7434AN4 chromosome was located at nt. 4,097,668–4,098,732 about 80 kb from the center toward the left end, which also contains 19 *dna*-like boxes (Figs S2 and 2).

TTA codons are rare in *Streptomyces* species due to their high G + C content (typically more than 70%); for example, TTA-bearing genes comprised 1.7 and 3.4% of the *S. coelicolor* and *S. avermitilis* genomes, respectively^22^. On the *S. rochei* 7434AN4 chromosome, 225 CDSs (2.9%) contain TTA codons (Table S4) (SRO_0031 and SRO_7538 are duplicates since they are in the TIR regions of the chromosome). Distribution of TTA-bearing CDSs in five *Streptomyces* genomes, *S. coelicolor*, *S. avermitilis*, *S. griseus*, *S. hygroscopicus*, *S. scabies*, and *S. rochei*, were analyzed by reciprocal BLAST-P search (Table S5). Among 225 TTA-bearing CDSs on the *S. rochei* chromosome, 182 (81%) are specific for *S. rochei* and 30 (13%) are shared with the closely related species *S*. *hygroscopicus*, suggesting that TTA-bearing CDSs are species-specific in *Streptomyces*. Since TTA, one of the six leucine codons, is rare in streptomycetes, *bldA*, a gene for UUA-specific tRNA, has a crucial role in morphological differentiation and antibiotic production^23–25^. Among 225 TTA-bearing CDSs on the *S. rochei* chromosome, 17 CDSs (yellow boxes in Table S4) are involved in secondary metabolite biosynthesis, including polyketide synthases and non-ribosomal peptide synthetases. In the pentamycin biosynthetic gene cluster (BGC) (Fig. S4), five of 12 CDSs (*pemA1*, *pemA2*, *pemA5*, *pemC*, and *pemR*) contain a TTA codon. On the contrary, the BGC for filipin (=14-deoxo-pentamycin) in *S. avermitilis* contains only one TTA-bearing CDS (*pteR*; a homolog of *pemR*), suggesting that pentamycin production in *S. rochei* is strictly controlled under *bldA*-dependent regulon.

### Comparative analysis of the *S. rochei* chromosome with the genomes of *S. coelicolor* A3(2), *S. avermitilis*, *S. griseus*, and *S. hygroscopicus*

Most *Streptomyces* species, including *S. coelicolor* A3(2), *S. avermitilis*, and *S. griseus*, have six *rrn* operons (16S-23S-5S), however, *S. rochei* 7434AN4 has seven *rrn* operons (Table 1). Unusual number of *rrn* operons were also reported for *S. albus* J1074 (seven operons)^26^ and for *S. xiamenensis* 318 (five operons)^27^. As shown in Fig. S3, six *rrn* operons (*rrnA*, *rrnC*, *rrnD*, *rrnE*, *rrnF*, and *rrnG*) in *S. rochei* are located between the highly conserved ORFs; for example, *rrnA* operon between putative beta-lactamase (SRO_2104; upstream of 16S) and putative aminotransferase (SRO_2103; downstream of 5S), and *rrnG* between phosphoenolpyruvate-dependent sugar phosphotransferase (SRO_6100; upstream of 16S) and CDP-alcohol phosphatidyltransferase (SRO_6101; upstream of 5S). The boundary regions around the seventh *rrn* operon, designated as *rrnB* in *S. rochei* (SRO_2782 at upstream of 16S, and SRO_2781 at upstream of 5S) are apparently different from those in *S. albus* J1074.

**Figure 4:**
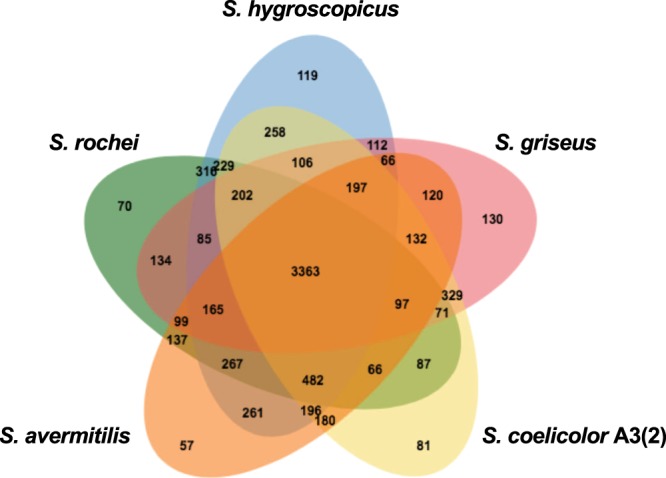
Venn diagram of the number of shared and unique genes between *S. rochei* and four other *Streptomyces* strains. OrthoVenn, a web-based application (http://www.bioinfogenome.net/OrthoVenn/) was used in this analysis. Other *Streptomyces* strains used in this analysis are *S. coelicolor* A3(2), *S. avermitilis* MA-4680, *S. griseus* IFO13350, and *S. hygroscopicus* 5008.

We then compared all CDSs on the chromosome of five *Streptomyces* strains, *S. coelicolor* A3(2), *S. avermitilis*, *S. griseus*, *S. hygroscopicus*, and *S. rochei*, using two independent *in silico* analyses; (1) orthologous clustering analysis by OrthoVenn Analysis Software (http://www.bioinfogenome.net/OrthoVenn/)^28^ and (2) pair-wise genome alignments by GenomeMatcher, a graphical interface for comparative genomics^29^. In ortholog clustering analysis, the CDSs on the *S. rochei* chromosome were classified into 5,370 clusters, among which 3,363 orthologs (44.4%) were shared in 5 strains (Fig. 4). In pair-wise genome alignments, the *S. rochei* chromosome contains a highly conserved core region (around nt 1.31–6.83 Mb) compared with the four reference strains (Fig. S5). However, a large genomic inversion was detected in *S. rochei*. When compared with *S. avermitilis*, a 1.54 Mb inversion was observed at 3.34–4.88 Mb region of the *S. rochei* chromosome, which corresponds to the 6.08–4.31 Mb region of the *S. avermitilis* chromosome.

### Biosynthetic gene clusters (BGCs) for secondary metabolites

We have focused on secondary metabolite biosynthetic machineries and regulatory pathways coded on pSLA2-L^4,11,30–33^. Several mutations including regulatory genes and major biosynthetic pathways coded on pSLA2-L led to activation of “silent” secondary metabolite clusters for pentamycin, citreodiol, *epi*-cireodiol, and KA57-A (Fig. 1)^34–36^. However, none of these biosynthetic gene clusters were found on pSLA2-L, suggesting their presence on the chromosome. Based on the 2ndFind database search (http://biosyn.nih.go.jp/2ndFind/) together with antiSMASH platform^37^, 35 BGCs for secondary metabolites were predicted on the *S. rochei* chromosome. These BGCs were classified into the following groups (Table 3); 8 for polyketides (PKSs), 8 for non-ribosomal peptides (NRPSs), 3 for hybrid PKS/NRPSs, 3 for lantibiotics, 5 for terpenes, 3 for siderophores, 1 for azoxyalkene, 1 for pseudosugar, 1 for butyrolactone, 1 for melanine, and 1 for ectoine (Table 3). The total length of BGCs (588 kb) occupied 7.03% of the *S. rochei* chromosome, which is comparable to other *Streptomyces* strains (594 kb and 6.6% for *S. avermitilis*).

**Table 3:** List of secondary metabolite biosynthetic gene clusters in the *S. rochei* chromosome.

Cluster^a^	Product type	Location	Length (bp)	Possible metabolites
1	PKS-NRPS	SRO_0155-SRO_0157	12,171	
2	Siderophore	SRO_0365-SRO_0370	8,474	
3	Terpene	SRO_0471-SRO_0472	2,153	2-Methylisoborneol
4	PKS-NRPS	SRO_0501-SRO_0517	49,191	
5	PKS	SRO_0730-SRO_0739	15,137	
6	Lantipeptide-NRPS	SRO_0832-SRO_0848	34,433	
7	PKS-NRPS	SRO_1003-SRO_1008	13,813	
8	NRPS-Lantipeptide	SRO_1184-SRO_1189	11,055	
9	Terpene	SRO_1200-SRO_1204	6,711	Hopene
10	NRPS	SRO_1300-SRO_1346	70,044	
11	NRPS	SRO_1551-SRO_1557	21,989	
12	PKS-NRPS	SRO_1613-SRO_1615	11,979	
13	Terpene	SRO_1735-SRO_1737	4,337	Geosmin
14	Azoxyalkene and type I PKS	SRO_1819-SRO_1850	36,010	KA57-A
15	Siderophore	SRO_2036-SRO_2038	4,744	
16	NRPS	SRO_2051-SRO_2053	5,458	
17	NRPS	SRO_2500-SRO_2506	9,881	
18	Terpene	SRO_2585-SRO_2586	2,410	Albaflavenone
19	Butyrolactone	SRO_3382	932	
20	NRPS-Lantipeptide	SRO_3845-SRO_3849	6,875	
21	Type II PKS	SRO_4037-SRO_4044	7,077	Spore pigment
22	Siderophore	SRO_4684-SRO_4688	5,997	
23	Melanin	SRO_4761-SRO_4762	1,446	Melanin
24	Ectoine	SRO_5592-SRO_5595	3,165	5-Hydroxyectoine
25	Type I PKS	SRO_6166-SRO_6168	12,248	
26	Type III PKS	SRO_6250-SRO_6251	2,284	
27	NRPS	SRO_6287-SRO_6291	9,037	
28	NRPS	SRO_6304-SRO_6312	46,390	
29	Type I PKS (Iterative)	SRO_6377-SRO_6381	12,789	
30	Pseudosugar	SRO_6636-SRO_6646	12,173	
31	NRPS	SRO_7209-SRO_7215	44,813	
32	Type I PKS	SRO_7221-SRO_7235	81,482	Pentamycin
33	Type I PKS (Iterative)	SRO_7329-SRO_7331	9,456	
34	NRPS	SRO_7379-SRO_7381	3,029	
35	Terpene	SRO_7452-SRO_7458	8,979	Carotenoid

^a^Secondary metabolite gene clusters were predicted by 2^nd^ Find and antiSMASH. The range of gene clusters for unknown secondary metabolites was estimated on the basis of putative functions of gene products.

Some chromosome-borne metabolites were obtained by genome mining on *S. rochei*^36^. The *lkcA* mutant (*lkcA*; an NRPS-PKS hybrid gene involved in lankacidin biosynthesis) overproduced three UV-active compounds, pentamycin, citreodiol, and *epi*-citreodiol (Fig. 1)^34^. Comparison of the pentamycin cluster (*pem*) with the filipin (=14-deoxypentamycin) cluster (*pte*) of *S. avermitilis*^38^ revealed their high homology (79–92% identities in ORFs) (Fig. S4), except for two additional genes, a P450 monooxygenase gene *SRO_7222* (*pemI*) and a ferredoxin gene *SRO_7221* (*pemJ*), in the former. At this stage, we have not yet identified the biosynthetic gene cluster for citreodiol and *epi*-citreodiol (*ctr* cluster). According to the reported feeding experiment^39^, the *ctr* cluster might include a C-methyltransferase (C-MT) gene for introduction of two methyl groups at C-2 and C-6. Two iterative type-I PKSs containing a C-MT domain (SRO_6380 and SRO_7330) are potential candidates for biosynthesis of citreodiols, whose gene inactivation is in progress in our laboratory. We isolated an azoxyalkene compound KA57-A (Fig. 1) from a genetically engineered strain KA57, which contains triple mutations on *srrB* (a *tetR*-type receptor gene), *lkcF-KR1* (a ketoreductase domain 1 of *lkcF* for lankacidin biosynthesis), and *lkmE* (a type-II thioesterase gene for lankamycin biosynthesis) coded on pSLA2-L^35^. KA57-A has a unique azoxy group (N = N^+^-O^−^) and its biosynthetic gene (*azx*) cluster was located at nt 2,061,273–2,097,283 of the chromosome by comparison with the BGC for valanimycin^40^. It is noteworthy that production of these four metabolites coded on the chromosome was activated by mutation of the genes coded on pSLA2-L, indicating that the linear plasmid affects not only on a topology of the linear chromosome but also on secondary metabolite production in *S. rochei*.

*Streptomyces* species produce branched-chain fatty acids for both primary and secondary metabolism^41^. Branched-chain amino acids, isoleucine, valine, and leucine, are converted to the corresponding 2-oxoacids, which were then decarboxylated to form 2-methylbutyryl-CoA, isobutyryl-CoA, and isovaleryl-CoA, respectively, by the branched-chain 2-oxoacid dehydrogenase complex (Fig. S6). In the biosynthesis of *Streptomyces* signaling molecules^42^, branched-chain β-ketoacyl-CoA esters (C_8_-C_13_ in length) are condensed with a dihydroxyacetone phosphate unit by specific enzyme such as AfsA (in *S. griseus*), ScbA (in *S. coelicolor*), BarX (in *S. virginiae*), and SrrX (in *S. rochei*). In *S. rochei*, the branched-chain fatty acid starter units for SRB1 and SRB2 (Fig. 1) are isobutyrate and (*S*)-2-methylbutyrate, respectively. Furthermore, the macrolide skeleton of lankamycin (Fig. 1) is also derived from one (*S*)-2-methylbutyrate starter unit and six malonyl-CoA extender units. The biosynthetic gene cluster *bkdFGH* for isobutyrate and (*S*)-2-methylbutyrate^43^ was also found on the *S. rochei* chromosome (SRO_3599, 3598, and 3597 for *bkdF, G and H*, respectively) (Fig. S6).

### Other genes

Comparative analysis of protein families with other four *Streptomyces* genome (*S. coelicolor* A3(2), *S. avermitilis*, *S. griseus*, and *S. hygroscopicus*) (Table S6) revealed that *S. rochei* 7434AN4 has relatively larger proportions of two-component histidine kinase gene homologs (113 vs 58 on average) and ABC transporter-related genes (338 vs 220 on average), reflecting a great extent of signal transduction and material transport. Strain 7434AN4 harbors 36 sigma factors and 21 ECF sigma factors. Homologous gene encoding the principal sigma factor σ^hrdB^ ^44^ was identified as *SRO_2011*. Two sporulation-related sigma factors, σ^BldN/AdsA^ for aerial mycelium formation in *S. coelicolor* A3(2)/*S. griseus*^45,46^ and σ^WhiG^ for onset of spore formation in *S. coelicolor* A3(2)^47^, were also identified as *SRO_3261* and *SRO_2209*, respectively. In addition, other important genes, σ^R^ for response to oxidative stress in *S. coelicolor* A3(2)^48^ and σ^shbA^ for governing σ^hrdB^ in *S. griseus*^49^ were also identified as *SRO_2590* and *SRO_2981*, respectively.

CRISPR (clustered regularly interspaced short palindromic repeats) is an RNA-dependent immune system widely distributed in bacteria and archaea against infection of foreign genetic elements including phages and plasmids^50^. The CRISPR-associated genes (*cas* genes) were identified from SRO_1948 to SRO_1955 (Table S7). Flanking this cluster, 19 DNA repeats (CGGTTCACCTCCGCCTGCGCGGAGCGGAC; 29 bases) were located upstream at nt. 2,205,703–2,206,838 and 6 repeats were downstream at nt. 2,216,850–2,217,183 (Table S8). All the Cas proteins showed considerable similarity with annotated *cas* gene products in other *Streptomyces* strains. The linear plasmid pSLA2-M has 49 repeat sequences at the right end of the CRISPR cluster (ORF94-ORF101)^12^, however, whose consensus sequences, 5′-GTGGCGGTCGCCCTCCGGGGTGACCGAGGATCGCAAC-3′ (37 bases), are different from that on the chromosome.

### Analysis of three plasmidless mutants, *S. rochei* 2-39, YN-P7, and YN-P145

**Figure 5:**
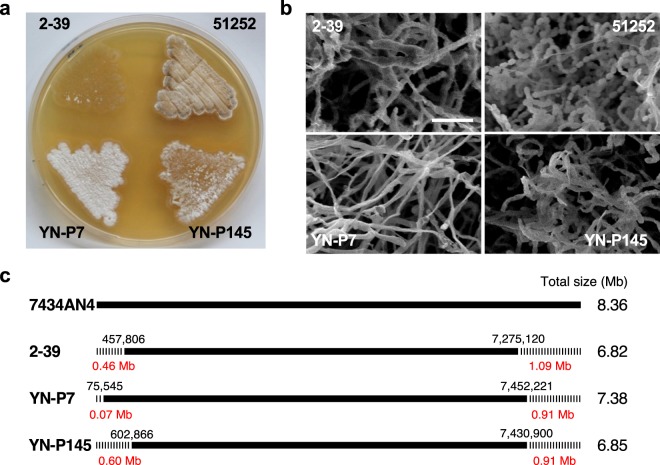
Morphological differentiation of three plasmidless mutants of *S. rochei* and their chromosomal deletion. (**a**) Spore formation of *S. rochei* strains. Strains (2-39, YN-P7, YN-P145, and their parent 51252) were grown on YM agar medium at 28 °C for 5 days. (**b**) Scanning electron microscopy (SEM) of surface grown colonies. (**c**) Chromosomal deletions in mutants 2-39, YN-P7, and YN-P145.

We previously prepared three plasmidless mutants, 2-39, YN-P7, and YN-P145 by protoplast regeneration of *S. rochei* 51252 that contains only pSLA2-L^9,14^. In the case of mutant 2-39, chromosomal deletion at both ends followed by circularization was confirmed by cloning of the fusion junction^14^. Although mutant 2-39 lost around 20% (1.55 Mb) of the chromosome (Fig. 5), seven rRNA operons were still conserved. PCR amplification showed that strains YN-P7 and YN-P145 also keep seven *rrn* operons, suggesting the importance of all *rrn* operons in *S. rochei*. Three mutants exhibited a different phenotype when grown on YM solid medium (Fig. 5); mutants YN-P7 and YN-P145 showed a “white” phenotype, while strain 2-39 a “bald” phenotype (Fig. 5a). To analyze morphological differentiation more precisely, their colonies were observed by scanning electron microscopy (SEM) (Fig. 5b). Chain elongation of aerial mycelium of mutant 2-39 stopped at an early stage, while mutant YN-P7 produced longer but collapsed hyphae. On the other hand, mutant YN-P145 produced partially spiral spore chains although their development is significantly lesser than that in strain 51252. Based on the Illumina sequence data of the three mutants, the bald strain 2-39 was found to suffer a larger chromosomal deletion from the right end (1,090 kb) compared with strains YN-P7 (913 kb) and YN-P145 (934 kb) (Fig. 5c). On the other hand, the deletions at the left chromosomal end of strains 2-39, YN-P7, and YN-P145 were 458 kb, 76 kb, and 603 kb, respectively. Based on the phenotype-genotype correlation in strains 2-39, YN-P7 and YN-P145, we speculate that essential gene(s) responsible for converting vegetative hyphae into aerial hyphae is(are) located at nt 7,275–7,431 kb of the 7434AN4 chromosome. This region harbors 138 ORFs (SRO_6607-SRO_6744), among which some gene(s) may be responsible for aerial hyphae formation in *S. rochei*.

## Conclusion

In this study, we have determined the nucleotide sequence of the 8,364,802-bp linear chromosome of *S. rochei* 7434AN4, which in turn revealed the gross genome size (8,706,406 bp) of this strain including the three linear plasmids, pSLA2-L, -M, and -S. General features of the linear chromosome were presented; it carries seven *rrn* operons, 67 tRNA genes, 225 TTA-containg CDSs, and 53.9-kb TIRs at both ends. In particular, the absence of a *tpg-tap* gene pair on the chromosome has proved our hypothesis that the *tpg-tap* pairs of pSLA2-L and/or pSLA2-M function to maintain a linear topology of the chromosome in strain 7434AN4.

*In silico* analysis indicated the presence of 35 secondary metabolites gene clusters on the chromosome, whose functions are not known in most cases. Therefore, we could expect that studies on their functions and regulation, particularly interaction with the regulatory genes coded on pSLA2-L will lead to a discovery of new antibiotics and their improved production.

## Materials and Methods

### Strains, plasmids, oligonucleotides, and culture media

All the strains, plasmids, and oligonucleotides used in this study were listed in Table S1. YEME liquid medium (0.3% yeast extract, 0.5% peptone, 0.3% malt extract, 1.0% D-glucose, 34% sucrose, 5 mM MgCl_2_, and 0.5% glycine) was used for preparation of total genomic DNA. YM medium (0.4% yeast extract, 1.0% malt extract, and 0.4% D-glucose, pH 7.3) was used for routine cultivation.

### DNA sequencing and assembly

*S. rochei* 7434AN4 was sequenced using hybrid approach of next-generation sequencing platforms; PacBio RS-II, Illumina GAIIx, and Roche 454 sequencers.

Genomic DNA of strain 7434AN4 was subjected to paired-end sequencing using Illumina GAIIx sequencing system (San Diego, CA, USA) according to the manufacture’s protocol. The 26,119,215 trimmed reads with 217-fold coverage of the whole genome were assembled using ABySS 1.3.7^51^. Illumina read data has been deposited as DRA Accession DRA003131 and DRA003132, Bioproject: PRJDB3565, Biosample: SAMD00027156. Independently, long-read sequencing was performed on PacBio RS-II sequencing system (Pacific Biosciences; Menlo Park, CA, USA). The filtered subreads with 598,149,379 bp in length (69-fold coverage) from PacBio RS-II was then assembled using the Hierarchical Genome Assembly Process (HGAP). The assembly consists of 175 contigs of 8,204,607 bp with an average length of 46,883 bp. Both sequence data was extensively compared and corrected with a help of Roche 454 pyrosequencing to obtain eight contigs (Fig. 2; length of contigs 1-8 were 2,393,094 bp, 1,685,790 bp, 1,286,111 bp, 750,875 bp, 720,184 bp, 567,335 bp, 501,529 bp, and 462,928 bp, respectively. Details for Roche 454 pyrosequencing were described previously^14^. Sequence gaps among eight contigs were then religiously filled and connected by conventional PCR amplification. Complete nucleotide sequence of the *S. rochei* 7434AN4 chromosome has been deposited at DDBJ under Accession number AP018517.

### Sequence annotation and comparative analysis

Putative coding sequences (CDSs), tRNA-, and rRNA-coding sequences were predicted using Microbial Genome Annotation Pipeline (MiGAP) platform (https://www.migap.org/) and FramePlot 2.3.2 (http://www0.nih.go.jp/~jun/cgi-bin/frameplot.pl)^52^. Their putative annotation was manually confirmed by a BLASTP program (https://blast.ncbi.nlm.nih.gov/Blast.cgi?PAGE=Proteins). Main features including distribution of rDNA operons, tRNAs, BGCs, and CDSs according to direction of transcription (+ strand, upper line; − strand, lower line) as well as GC-skew diagram (Fig. 2) were generated by DNA plotter software (https://www.sanger.ac.uk/science/tools/dnaplotter)^15^. The locus of *oriC* was predicted manually based on the genome information of other *Streptomyces* species. The protein families were clustered with OrthoVenn Analysis Software (http://www.bioinfogenome.net/OrthoVenn/), a web platform for comparison and annotation of orthologous gene clusters among multiple species^28^. The comparative analysis of the chromosomes between *S. rochei* 7434AN4 and other *Streptomyces* species was performed using GenomeMatcher software (http://www.ige.tohoku.ac.jp/joho/gmProject/gmhomeJP.html)^29^ and bl2seq program, which is embedded in the application bundled. Secondary metabolite gene clusters were predicted by either 2ndFind software, a web-based analytical tool (http://biosyn.nih.go.jp/2ndFind/), or antiSMASH 2.0, a web-based analysis platform (http://antismash.secondarymetabolites.org/)^37^. CRISPRs were predicted using a CRISPRFinder (http://crispr.i2bc.paris-saclay.fr/Server/), an online program.

### DNA manipulation and Southern hybridization

*Streptomyces* strains were grown in liquid YM medium in Sakaguchi flask at 28 °C for 3 days. DNA manipulation of *Streptomyces* species^53^ was carried out according to standard procedure. Genome DNA sample of *S. rochei* 7434AN4 for PFGE was obtained according to the method as described previously^9^ with a slight modification. Polymerase chain reaction (PCR) was performed on a GeneAtlas G02 Thermal Cycler (Astec Co. Ltd., Fukuoka, Japan) using KOD-plus Neo DNA polymerase (Toyobo, Osaka, Japan) according to the manufacture’s protocol. Southern blot analysis (Figs 3, 5 and S2) was performed as described previously^14^.

### Scanning electron microscopy (SEM)

The surface morphology of *S*. *rochei* strain 51252 and three plasmidless mutants was observed by scanning electron microscopy (SEM) after growing on YM agar plate for 5 days. For the preparation of specimens, agar plugs were fixed with 1% osmium tetroxide solution for 12 h, and then dehydrated by lyophilization. The resulting specimens were coated with platinum (2 nm) and observed by a Jeol JSM-5900 Scanning Electron Microscope.

## Supplementary information

Supplementary materials
