# Supplementary material for: The genome sequence of Streptomyces rochei 7434AN4, which carries a linear chromosome and three characteristic linear plasmids
Source: Sci Rep. 2019 Jul 29;9:10973. doi: 10.1038/s41598-019-47406-y (PMC6662830; doi:10.1038/s41598-019-47406-y)
Supplement: Supplementary file 1 — Supplementary materials [file 41598_2019_47406_MOESM1_ESM.pdf]

Supplementary materials

The genome sequence of *Streptomyces rochei* 7434AN4, which carries a linear chromosome and three characteristic linear plasmids

Yosi Nindita,[1,2] Zhisheng Cao,[1] Amirudin Akhmad Fauzi,[1] Aiko Teshima,[1] Yuya Misaki,[1,2] Rukman Muslimin,[1] Yingjie Yang,[1] Yuh Shiwa,[3,4] Hirofumi Yoshikawa,[4] Michihira Tagami,[5] Alexander Lezhava,[5] Jun Ishikawa,[6] Makoto Kuroda,[7] Tsuyoshi Sekizuka,[7] Kuninobu Inada,[8] Haruyasu Kinashi[1] & Kenji Arakawa[1,2*]

[1]Department of Molecular Biotechnology, Graduate School of Advanced Sciences of Matter, and [2]Unit of Biotechnology, Division of Biological and Life Sciences, Graduate School of Integrated Sciences for Life, Hiroshima University, 1-3-1 Kagamiyama, Higashi-Hiroshima 739-8530, Japan. [3]NODAI Genome Research Center and [4]Department of Bioscience, Tokyo University of Agriculture, 1-1-1 Sakuragaoka, Setagaya-ku, Tokyo 156-8502, Japan. [5]Omics Science Center, RIKEN, 1-7-22 Suehiro-cho, Tsurumi-ku, Yokohama, Kanagawa 230-0045, Japan. [6]Department of Bioactive Molecules and [7]Pathogen Genomics Center, National Institute of Infectious Diseases, 1-23-1 Toyama, Shinjuku-ku, Tokyo 162-8640, Japan. [8]Natural Science Center for Basic Research and Development, Hiroshima University, 1-4-2 Kagamiyama, Higashi-Hiroshima 739-8526, Japan.

* Correspondence should be addressed to K.A. (email: karakawa@hiroshima-u.ac.jp)

List of Supplementary materials

Supplementary Fig. S1
Nucleotide sequence of the *oriC*-franking region.

Supplementary Fig. S2
Genome size estimation of *S. rochei* 7434AN4 by pulsed-field gel electrophoresis (PFGE).

Supplementary Fig. S3
Comparison of *rrn* operons among *S. rochei* and other *Streptomyces* species.

Supplementary Fig. S4
Comparison of biosynthetic gene cluster for pentamycin in *S. rochei* with that for filipin in *S. avermitilis*.

Supplementary Fig. S5
Pair-wise genome comparison of the *S. rochei* chromosome with five reference *Streptomyces* strains.

Supplementary Fig. S6
Genes involved in branched-chain fatty acid biosynthesis pathway in *S. rochei* 7434AN4.

Supplementary Table S1
Bacterial strains and oligonucleotides used in this study.

Supplementary Table S2
Lists of rRNA operons in the *S. rochei* 7434AN4 chromosome.

Supplementary Table S3
Lists of tRNA genes in the *S. rochei* 7434AN4 chromosome.

Supplementary Table S4
List of TTA codon-containing ORFs in the *S. rochei* chromosome.

Supplementary Table S5

TTA-containing ORFs in the *S. rochei* chromosome classified by their occurrence in other *Streptomyces* species.

Supplementary Table S6
Protein families in *S. rochei* 7434AN4 and other four completely sequenced *Streptomyces.*

Supplementary Table S7
Location of CRISPR-associate (*cas*) genes and CRISPR repeats in the *S. rochei* chromosome.

Supplementary Table S8
Sequences of CRISPR repeats in the *S. rochei* chromosome.

a

b

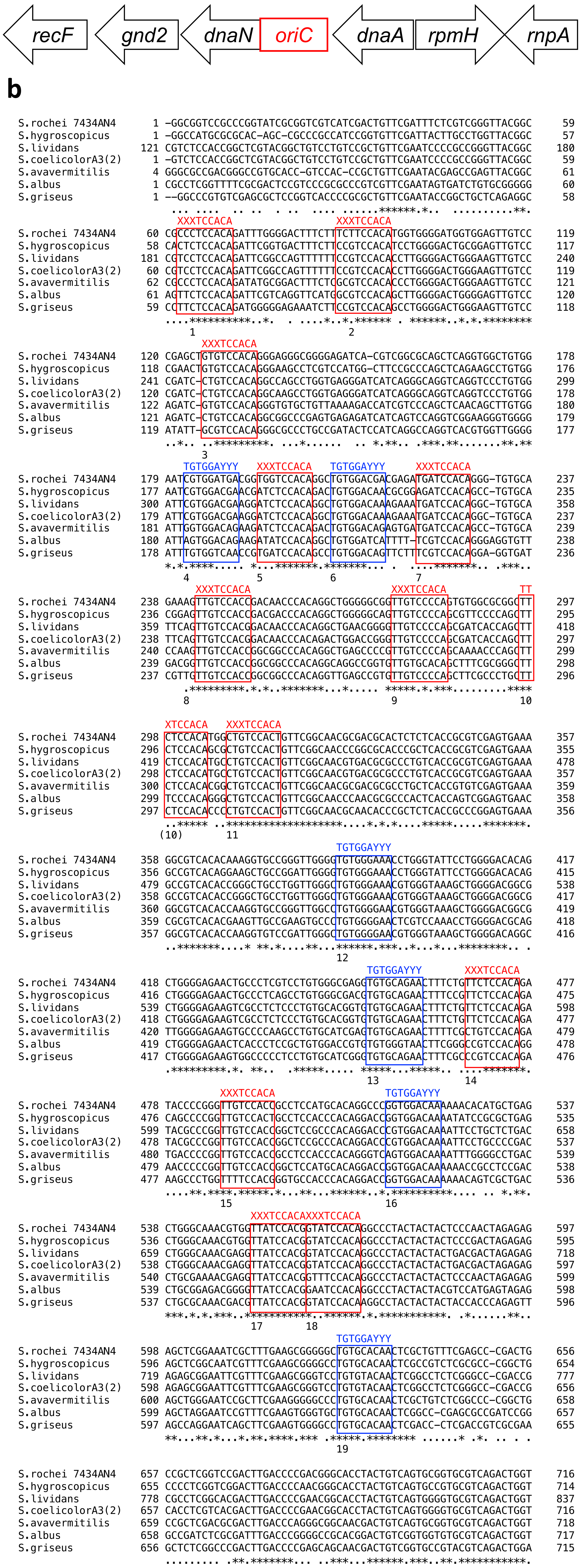

Figure S1. Nucleotide sequence of the *oriC*-franking region. (**a**) *oriC* and its flanking region of *S. rochei* 7434AN4, Putative *oriC* region of *S. rochei* 7434AN4 is shown in the interspace region between *dnaA* and *dnaN* region (nt 4,098,733 – 4,097,667). (**b**) Alignment of the nucleotide sequences of the *Streptomyces* putative *oriC* region (Query 7434AN4; nt 4,098,461- 4,097,683. Putative *dnaA* boxes are (T/C)(T/C)(G/A/C)TCCACA(direction →) and TGTGGA(C/T/G)(A/G)(A/G) (direction ←) (Microbiology, 144, 1281-1290 (1998)).

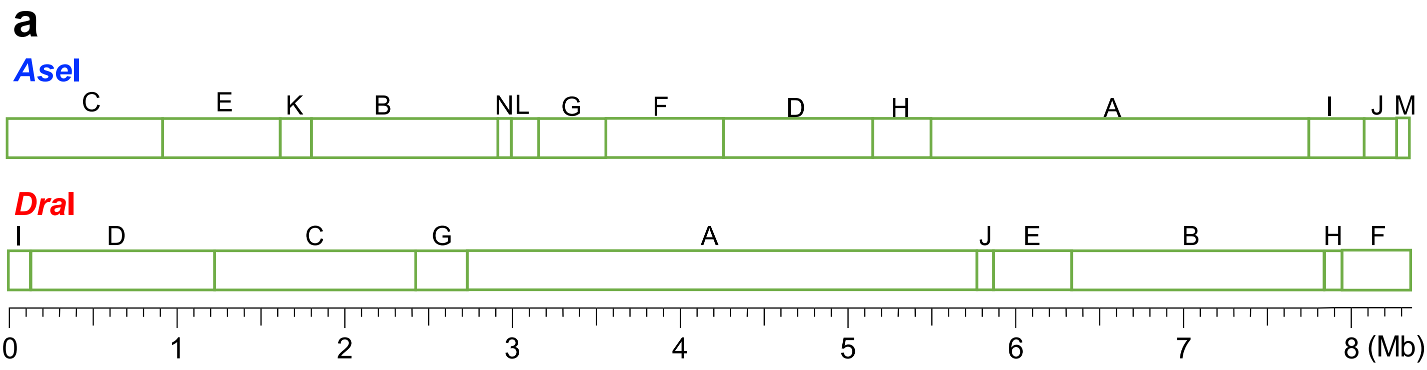

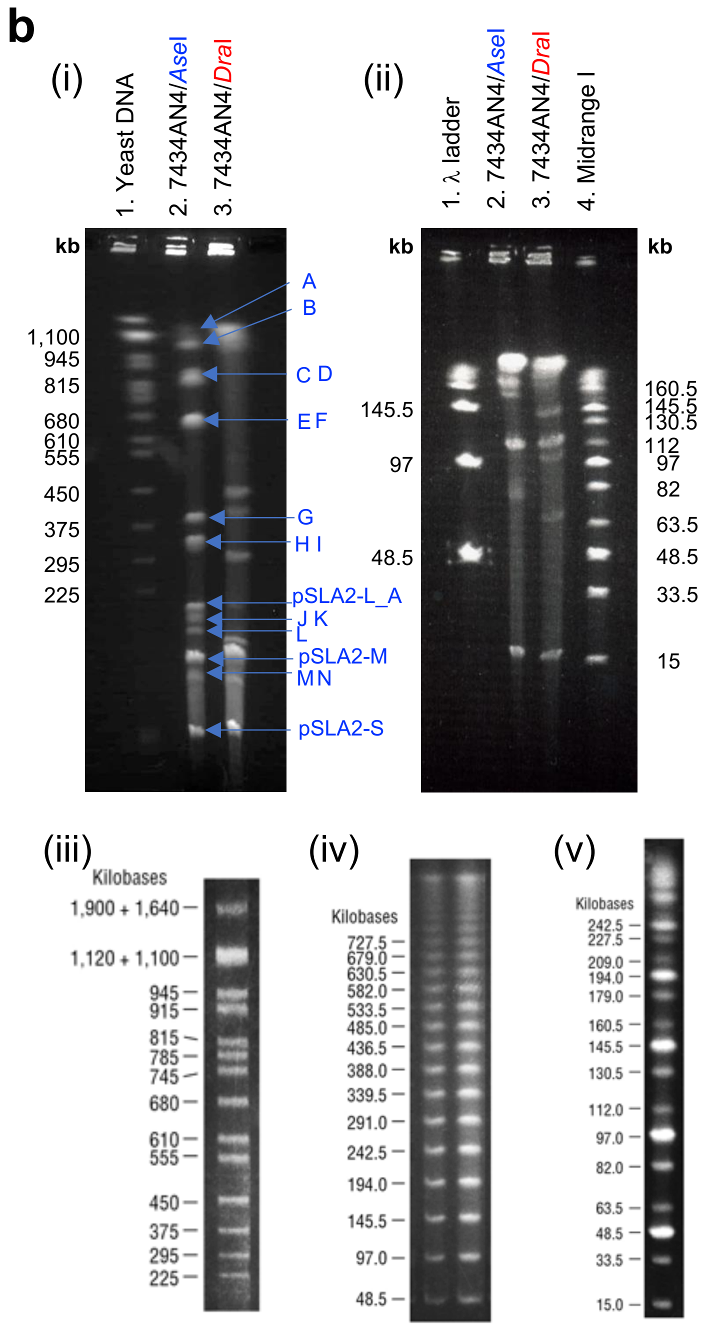

c

Fragment	*Ase*I (bp)	*Dra*I (bp)
A	2,249,923	3,042,329
B	1,110,729	1,520,514
C	926,337	1,192,836
D	889,703	1,092,359
E	700,374	473,607
F	699,913	416,878
G	400,833	307,059
H	345,559	111,971
I	333,248	104,652
J	192,864	102,597
K	187,063	-
L	164,803	-
M	83,833	-
N	79,620	-
pSLA2-L_ A	206,627	141,192
pSLA2-L_ B	3,987	69,422
pSLA2-M	113,463	113,463
pSLA2-S	17,526	17,526

Figure S2. Genome size estimation of *S. rochei* 7434AN4 by pulsed-field gel electrophoresis (PFGE). (**a**) Physical maps of *Ase*I- and *Dra*I-fragments of the *S. rochei* chromosome. (**b**) PFGE of the DNA samples prepared in gels and digested either *Ase*I or *Dra*I. (i) 1. Yeast chromosome PFG marker, 2. 7434AN4 total DNA digested with *Ase*I, 3. 7434AN4 total DNA digested with *Dra*I, (ii) 1. λ ladder pulsed-field gel (PFG) marker, 2. 7434AN4 total DNA digested with *Ase*I, 3. 7434AN4 total DNA digested with *Dra*I, 4. Midrange I PFG marker. (iii) Yeast chromosome PGF marker (New England Biolabs cat# N0345). (iv) λ ladder PFG marker (New England Biolabs cat# N0340). (v) Midrange I PFG marker (New England Biolabs cat# N3551). (**c**) Estimated DNA sizes of the *Ase*I- and *Dra*I-fragments of the *S. rochei* total DNA.

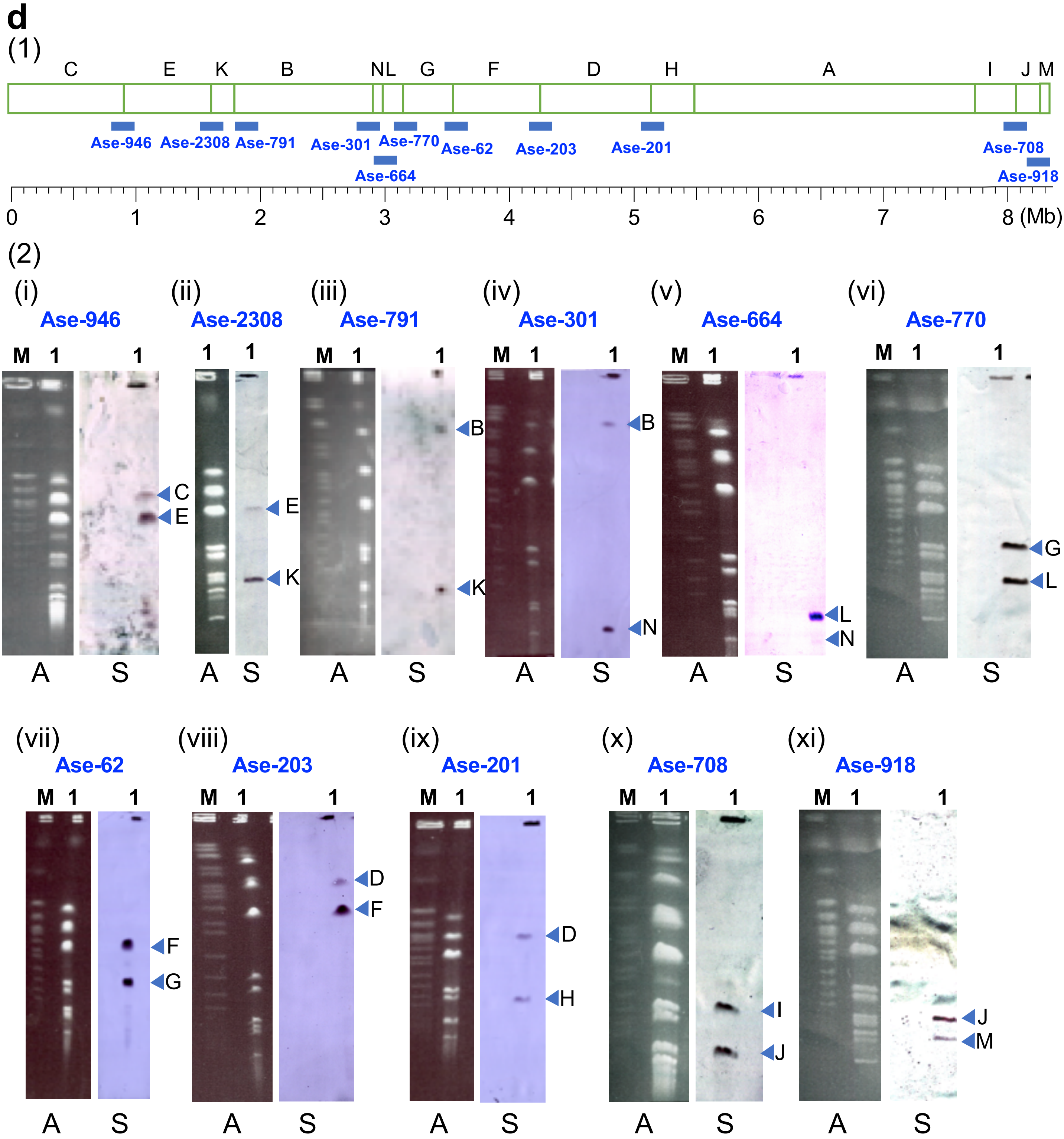

Figure S2 (continued). Genome size estimation of *S. rochei* 7434AN4 by pulsed-field gel electrophoresis (PFGE). (**d**) Confirmation of the location of *Ase*I-fragments in the *S. rochei* chromosome. (1) Physical map of *Ase*I-fragment of the *S. rochei* chromosome. Locations of DNA probe for Southern hybridization were also shown. (2) PFGE and Southern blotting images. Panels shown as "A" indicate PFGE image, while panels "S" indicate Southern blotting image. PFGE gels (panel "A") were hybridized with the following probes; (i) Ase-946 (PCR fragment between nt 925,564-927,697), (ii) Ase-2308 (nt 1,625,990-1,628,415), (iii) Ase-791 (nt 1,813,314-1,814,826), (iv) Ase-301 (nt 2,923,341-2,925,323), (v) Ase-664 (nt 3,003,812-3,006,047), (vi) Ase-770 (nt 3,167,813-3,170,278), (vii) Ase-62 (nt 3,568,362-3,570,807), (viii) Ase-203 (nt 4,268,134-4,270,234), (ix) Ase-201 (nt 5,158,768-5,161,337), (x) Ase-708 (nt 8,087,283-8,089,195), (xi) Ase-918 (nt 8,279,773-8,281,515). Lanes M, Yeast chromosome PFG marker; lanes 1, *S. rochei* total DNA digested with *Ase*I. Primers for probe preparation were shown in Table S1. We used same agarose gel sample for (vi) and (xi). Reprobing was performed in the analysis of Ase-918. First probe Ase-770 was stripped off from nylon membrane.

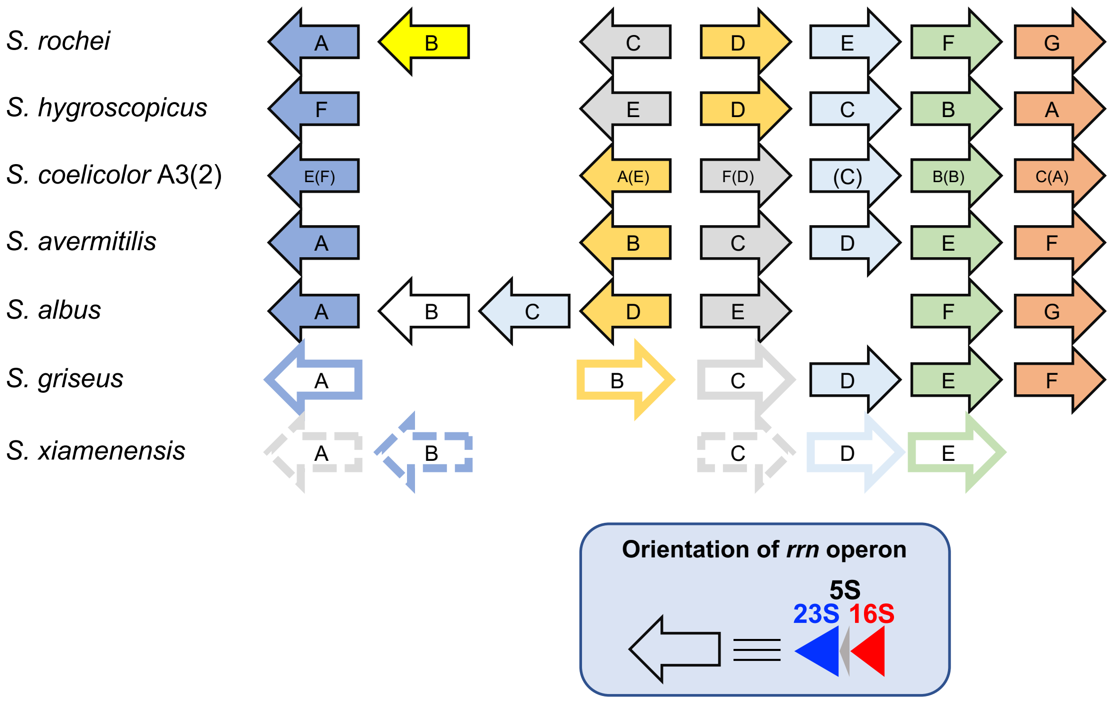

Figure S3. Comparison of *rrn* operons among *S. rochei* and other *Streptomyces* species.

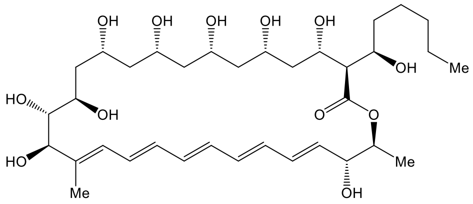

Pentamycin

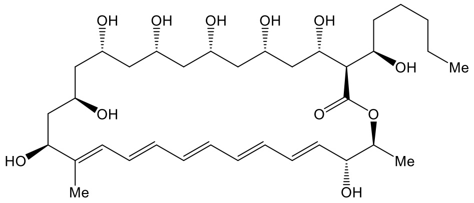

Filipin (=14-deoxypentamycin)

Filipin cluster (*S. avemitilis* nt 487,415-567,017)

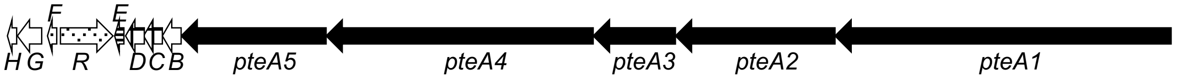

Pentamycin cluster (*S. rochei* nt 7,951,834-8,033,316)

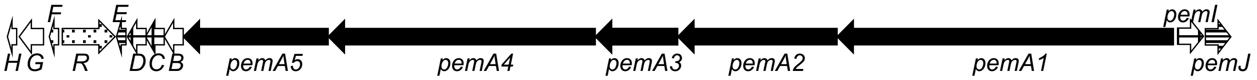

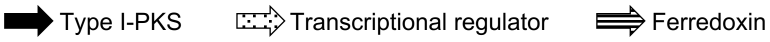

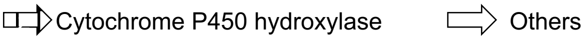

Filipin cluster (aa)	Pentamycin cluster (aa)	Identity (%)
pteA1 (7746)	SRO_7223; pemA1 (7807)	6440/7655 (84%)
pteA2 (3564)	SRO_7224; pemA2 (3598)	3107/3603 (86%)
pteA3 (1835)	SRO_7225; pemA3 (1821)	1556/1834 (84%)
pteA4 (6145)	SRO_7226; pemA4 (6155)	5228/6158 (84%)
pteA5 (3352)	SRO_7227; pemA5 (3353)	2873/3359 (85%)
pteB (418)	SRO_7228; pemB (418)	388/418 (92%)
pteC (399)	SRO_7229; pemC (399)	369/399 (92%)
pteD (404)	SRO_7230; pemD (404)	360/404 (89%)
pteE (64)	SRO_7231; pemE (64)	51/64 (79%)
pteR (1197)	SRO_7232; pemR (1189)	945/1182 (79%)
pteF (232)	SRO_7233; pemF (204)	180/204 (88%)
pteG (547)	SRO_7234; pemG (547)	492/545 (90%)
pteH (255)	SRO_7235; pemH (255)	207/255 (81%)
-	SRO_7222; pemI (405)	-
-	SRO_7221; pemJ (64)	-

Figure S4. Comparison of biosynthetic gene cluster for pentamycin in *S. rochei* 7434AN4 with that for filipin in *S. avermitilis.*

a

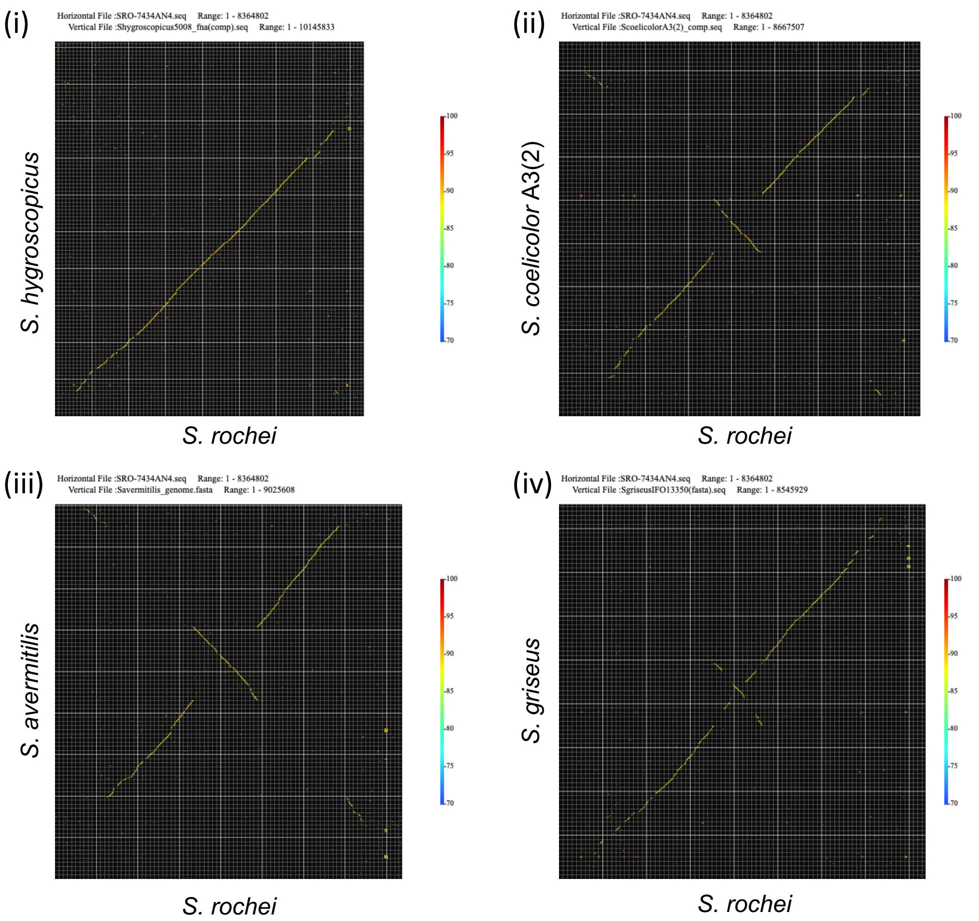

b

Reference strain	Conserved region in *S. rochei* and references	
	S. rochei	Reference strain
S. hygroscopicus	545,022-7,559,915	2,338,139-9,459,009
S. coelicolor A3(2)	1,312,392-7,187,111	1,052,906-7,520,687
S. avermitilis	1,280,438-6,892,333	1,938,250-8,456,079
S. griseus	1,305,391-6,830,535	1,047,678-7,484,966

Highly conserved core region in *S. rochei* ; nt 1.31-6.83 Mb

Figure S5. Pair-wise genome comparison of the *S. rochei* chromosome with five reference *Streptomyces* strains. (**a**) Dots represent reciprocal best matches. Horizontal axises are the *S. rochei* chromosome. Vertical axises are the chromosome of (i) *S. hygroscopicus*, (ii) *S. coelicolor* A3(2), (iii) *S. avermitilis*, and (iv) *S. griseus*. (**b**) Highly conserved region in *S. rochei* and four reference strains.

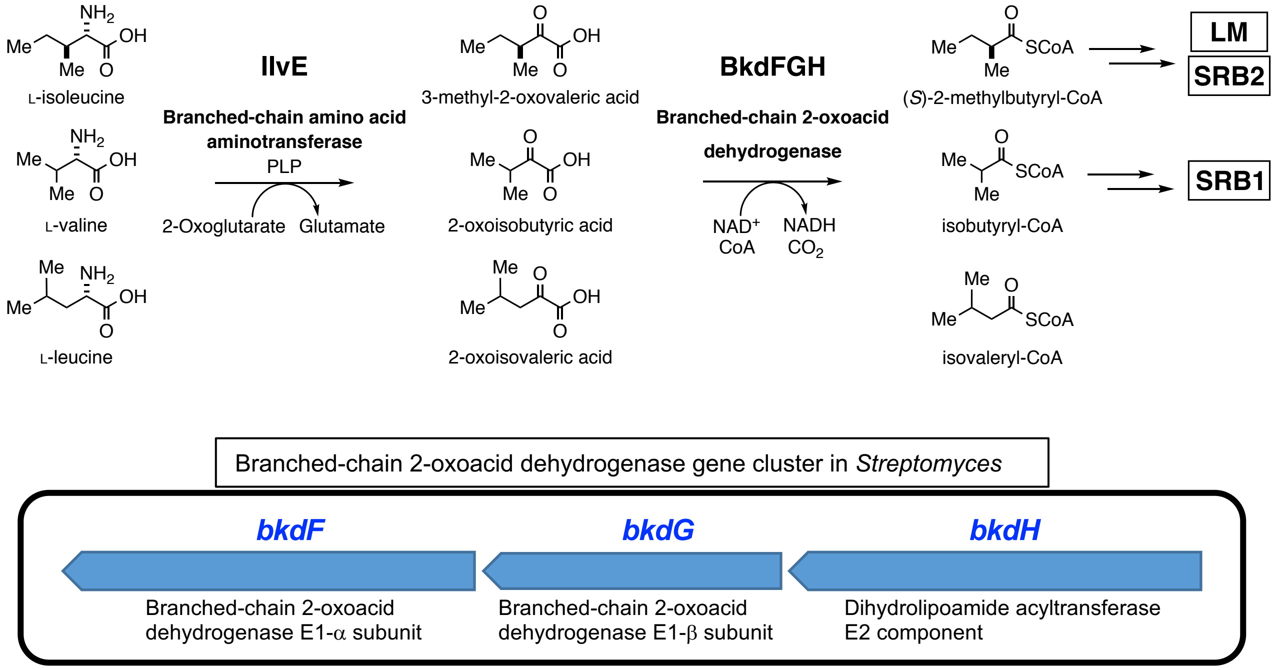

Fab enzyme	Proposed function	*S. rochei*		*S. avermitilis*	
		SRO_locus	Amino acid	SCO_locus	Amino acid
IlvE	Branched-chain amino acid transferase	SRO_2307	362	SAVERM_2717	362
BkdF	Branched-chain 2-oxoacid dehydrogenase E1-α subunit	SRO_3599	417	SAVERM_4376	406
BkdG	Branched-chain 2-oxoacid dehydrogenase E1-β subunit	SRO_3598	325	SAVERM_4377	325
BkdH	Dihydrolipoamide acyltransferase E2 component	SRO_3597	474	SAVERM_4378	462

Figure S6. Genes involved in branched-chain fatty acid biosynthesis pathway in *S. rochei* 7434AN4. 2-Methylbutyryl-CoA starter units (C14-C17 in LM and C7-C10,C-9’ in SRB2) are originated from L-isoleucine. Isobutyryl-CoA starter unit (C7-C9,C-9’ in SRB1) is originated from L-valine.

Table S1. Bacterial strains and oligonucleotides used in this study

Strains	Properties/product	Source/ref.
S. rochei		
7434AN4	Wild type (pSLA2-L,M,S)	Kinashi et al. 1994
51252	Ultraviolet irradiation of 7434AN4 (pSLA2-L)	Kinashi et al. 1994
2-39	Protoplast regeneration of 51252 (no plasmid)	Kinashi et al. 1994
YN-P7	Protoplast regeneration of 51252 (no plasmid)	Nindita et al. 2015
YN-P145	Protoplast regeneration of 51252 (no plasmid)	Nindita et al. 2015

Designed oligonucleotides	Nucleotide sequence	Region in the chromosome (purpose)
YN-TIR-f	5'-AAGAATTCCCTTCCACGCCTGGCGGCAC-3'	nt 52,079- (TIR detection)
YN-TIR-r	5'-TTAAGCTTCCCCTACCACGCCCCGGTCT-3'	nt 55,053- complement (TIR detection)
scf488-Anti	5'-TCCGCACACCCTTCAGCTTGGTGTCCAAGT-3'	nt 2,389,384- (franking region beyond *rrnA*-16S)
scf418-UP	5'-TGTGGGCGAGCCCGCGCTCAAGGACCGCAA-3'	nt 2,395,771- complement (franking region beyond *rrnA*-5S)
scf430-Anti	5'-GGGGGATTCTCTGGTCCCCTCTGGTAGCTA-3'	nt 3,141,102- (franking region beyond *rrnB*-16S)
scf585-UP	5'-CCGCCGCGCCCGGATAGTGGCCGACCACAA-3'	nt 3,147,450- complement (franking region beyond *rrnB*-5S)
scf584-Anti	5'-GGCCCGGTACAGCTCGAAGTGGGAGATGAA-3'	nt 3,639,770- (franking region beyond *rrnC*-16S)
scf416-UP	5'-GACGATCAACGCCATCCGCGAGCTGGAGAA-3'	nt 3,646,123- complement (franking region beyond *rrnC*-5S)
scf574-Anti	5'-GGTGCCGGGCACGGAGAACTGGGAGTACGT-3'	nt 4,365,140- complement (franking region beyond *rrnD*-16S)
scf413-UP	5'-GCTGGAGGCGATGCGCCTCGCGACCGTCTT-3'	nt 4,358,753- (franking region beyond *rrnD*-5S)
scf443-Anti	5'-GCTGGACGCGGCACCGGTGGAACAGGGACT-3'	nt 4,934,256- complement (franking region beyond *rrnE*-16S)
scf412-UP	5'-CCTGTTCGTCTCCGGCGCCATCGAAGGGTT-3'	nt 4,927,974- (franking region beyond *rrnE*-5S)
scf422-Anti	5'-CCGCCACGGTCGCCACCGCGGTCGTCATCA-3'	nt 6,219,749- complement (franking region beyond *rrnF*-16S)
scf417-UP	5'-GTTCGGGGAGCCCGGACATGTGTACGTCTA-3'	nt 6,212,930- (franking region beyond *rrnF*-5S)
scf439-Anti	5'-GGAGGATGCCCACCATCACCAGGAGCATCA-3'	nt 6,682,773- complement (franking region beyond *rrnG*-16S)
scf419-UP	5'-GCCGTGTCCGCTGTCGTCGACCACGACGAA-3'	nt 6,676,445- (franking region beyond *rrnG*-5S)
Ase-946f	5'-TTGAATTCGTCGTTGTCGTCATGATGTTCT-3'	nt 925,564- (boundary between AseI-C and AseI-E)
Ase-946r	5'-TTCTGCAGCTGTGTCTCAAACTACCCCACA-3'	nt 927,697- complement (boundary between AseI-C and AseI-E)
Ase-2308f	5'-TTGAATTCGTCGTCCGGGAGTTGAGTC-3'	nt 1,625,990- (boundary between AseI-E and AseI-K)
Ase-2308r	5'-TTCTGCAGGCAGGAGTTGACCGACAAGATA-3'	nt 1,628,415- complement (boundary between AseI-E and AseI-K)
Ase-791f	5'-TTGAATTCCACTCATGGGTGAGCACGGCAT-3'	nt 1,813,314- (boundary between AseI-K and AseI-B)
Ase-791r	5'-TTCTGCAGCGAATTCATACATGAGGGAGAA-3'	nt 1,814,826- complement (boundary between AseI-K and AseI-B)
Ase-301f	5'-TTGAATTCGAACCAGTAGACCGAGCTGAAC-3'	nt 2,923,341- (boundary between AseI-B and AseI-N)
Ase-301r	5'-TTGAATTCCTGTACACGCAGAATTTTCCAG-3'	nt 2,925,323- complement (boundary between AseI-B and AseI-N)
Ase-664f	5'-TTGAATTCGGTTGATGTTCTTCCTCTGGTC-3'	nt 3,006,047- complement (boundary between AseI-N and AseI-L)
Ase-664R	5'-TTGAATTCGTAGGCCACGCCCGTGAAGAGC-3'	nt 3,003,812 - (boundary between AseI-N and AseI-L)
Ase-770f	5'-TTGAATTCGTGCTTGCTCATCGACTTCAT-3'	nt 3,167,813- (boundary between AseI-L and AseI-G)
Ase-770r	5'-TTCTGCAGGAACTCCACCTGGAGACCAAG-3'	nt 3,170,278- (boundary between AseI-L and AseI-G)
Ase-62F	5'-TTGAATTCCAACATGTTCTCGGTGTTCTTC-3'	nt 3,570,807- complement (boundary between AseI-G and AseI-F)
Ase-62r	5'-TTGAATTCCCGCATTTCAGGAGCTATTTAC-3'	nt 3,568,362- (boundary between AseI-G and AseI-F)
Ase-203f	5'-TTGAATTCACCTCGCTCTTGAAAACTAACG-3'	nt 4,270,234- complement (boundary between AseI-F and AseI-D)
Ase-203r	5'-TTGAATTCCCTCTTGTGAGGTCTCGTCTCT-3'	nt 4,268,134- (boundary between AseI-F and AseI-D)
Ase-201f	5'-TTGAATTCTACATCAAGGAGCATGTGAAGG-3'	nt 5,158,768- (boundary between AseI-D and AseI-H)
Ase-201r	5'-TTGAATTCGCATGGTCTACGGCTAGGAATA-3'	nt 5,161,337- complement (boundary between AseI-D and AseI-H)
Ase-708f	5'-TTGAATTCTAGGACTGGAGCCTCTTGTAGG-3'	nt 8,089,195- complement (boundary between AseI-I and AseI-J)
Ase-708r	5'-TTCTGCAGGTCGAGCAGGCTTCTGAGATAC-3'	nt 8,087,283- (boundary between AseI-I and AseI-J)
Ase-918f	5'-TTGAATTCTTCGGCGATGTGGGGATGCAGT-3'	nt 8,279,773- (boundary between AseI-J and AseI-M)
Ase-918r	5'-TTCTGCAGGGTCAGCAGACGGTAGTAGAGG-3'	nt 8,281,515- complement (boundary between AseI-J and AseI-M)

Table S2. Lists of rRNA genes in the *S. rochei* 7434AN4 chromosome.

No.	Locus tag	Direction	Start	Stop	Product
rrnA	SRO_r01	–	2389969	2389854	*rrnA* 5S ribosomal RNA
	SRO_r02	–	2393162	2390043	*rrnA* 23S ribosomal RNA
	SRO_r03	–	2394974	2393447	*rrnA* 16S ribosomal RNA
rrnB	SRO_r04	–	3141720	3141605	*rrnB* 5S ribosomal RNA
	SRO_r05	–	3144914	3141794	*rrnB* 23S ribosomal RNA
	SRO_r06	–	3146761	3145234	*rrnB* 16S ribosomal RNA
rrnC	SRO_r07	–	3640341	3640226	*rrnC* 5S ribosomal RNA
	SRO_r08	–	3643534	3640415	*rrnC* 23S ribosomal RNA
	SRO_r09	–	3645371	3643844	*rrnC* 16S ribosomal RNA
rrnD	SRO_r10	+	4359548	4361075	*rrnD* 16S ribosomal RNA
	SRO_r11	+	4361387	4364507	*rrnD* 23S ribosomal RNA
	SRO_r12	+	4364581	4364696	*rrnD* 5S ribosomal RNA
rrnE	SRO_r13	+	4928726	4930253	*rrnE* 16S ribosomal RNA
	SRO_r14	+	4930538	4933655	*rrnE* 23S ribosomal RNA
	SRO_r15	+	4933729	4933844	*rrnE* 5S ribosomal RNA
rrnF	SRO_r16	+	6213883	6215410	*rrnF* 16S ribosomal RNA
	SRO_r17	+	6215713	6218833	*rrnF* 23S ribosomal RNA
	SRO_r18	+	6218941	6219056	*rrnF* 5S ribosomal RNA
rrnG	SRO_r19	+	6677172	6678699	*rrnG* 16S ribosomal RNA
	SRO_r20	+	6678984	6682103	*rrnG* 23S ribosomal RNA
	SRO_r21	+	6682177	6682292	*rrnG* 5S ribosomal RNA

Table S3. Lists of tRNA genes in the *S. rochei* 7434AN4 chromosome.

No.	Locus tag	Direction	Start	Stop	Product
1	SRO_t01	–	1233129	1233056	tRNA-Pro
2	SRO_t02	–	1251754	1251681	tRNA-Pro
3	SRO_t03	–	1290129	1290056	tRNA-Pro
4	SRO_t04	–	1519798	1519711	tRNA-Leu
5	SRO_t05	–	2388964	2388892	tRNA-Thr
6	SRO_t06	–	2585963	2585891	tRNA-Glu
7	SRO_t07	–	2586064	2585993	tRNA-Gln
8	SRO_t08	–	2586157	2586082	tRNA-Glu
9	SRO_t09	–	2586259	2586187	tRNA-Glu
10	SRO_t10	–	2586352	2586281	tRNA-Gln
11	SRO_t11	+	2821384	2821458	tRNA-Arg
12	SRO_t12	–	2937912	2937839	tRNA-Met
13	SRO_t13	–	2940126	2940053	tRNA-Met
14	SRO_t14	–	3449917	3449842	tRNA-Trp
15	SRO_t15	–	3459734	3459662	tRNA-Met
16	SRO_t16	–	3459853	3459781	tRNA-Thr
17	SRO_t17	–	3466004	3465923	tRNA-Tyr
18	SRO_t18	–	3766257	3766184	tRNA-Thr
19	SRO_t19	+	3819429	3819502	tRNA-Pro
20	SRO_t20	+	3904972	3905045	tRNA-Gly
21	SRO_t21	–	4057244	4057161	tRNA-Leu
22	SRO_t22	–	4074213	4074141	tRNA-Ala
23	SRO_t23	–	4087738	4087662	tRNA-Ile
24	SRO_t24	+	4196100	4196186	tRNA-Ser
25	SRO_t25	+	4210330	4210423	tRNA-Ser
26	SRO_t26	+	4210601	4210673	tRNA-Arg
27	SRO_t27	–	4234750	4234666	tRNA-Ser
28	SRO_t28	–	4292090	4292003	tRNA-Ser
29	SRO_t29	–	4301285	4301210	tRNA-Gly
30	SRO_t30	–	4305226	4305151	tRNA-Gly
31	SRO_t31	–	4318725	4318651	tRNA-Asp
32	SRO_t32	–	4324459	4324383	tRNA-Phe
33	SRO_t33	–	4324558	4324484	tRNA-Asp
34	SRO_t34	–	4324670	4324598	tRNA-Glu
35	SRO_t35	+	4331524	4331596	tRNA-Lys
36	SRO_t36	+	4335773	4335846	tRNA-Met
37	SRO_t37	+	4473327	4473403	tRNA-Thr
38	SRO_t38	–	4685066	4684980	tRNA-Arg
39	SRO_t39	–	4714739	4714664	tRNA-Arg
40	SRO_t40	+	4747834	4747907	tRNA-Ala
41	SRO_t41	–	4805439	4805365	tRNA-Gln
42	SRO_t42	+	4848715	4848801	tRNA-Leu
43	SRO_t43	–	5026162	5026088	tRNA-Cys
44	SRO_t44	+	5077321	5077396	tRNA-Leu
45	SRO_t45	–	5119361	5119285	tRNA-Lys
46	SRO_t46	–	5123002	5122926	tRNA-Lys
47	SRO_t47	–	5134228	5134155	tRNA-Lys
48	SRO_t48	+	5159209	5159281	tRNA-His
49	SRO_t49	–	5251955	5252030	tRNA-Arg
50	SRO_t50	–	5332137	5332207	tRNA-Gly
51	SRO_t51	+	5332368	5332444	tRNA-Pro
52	SRO_t52	+	5380478	5380550	tRNA-Ala
53	SRO_t53	+	5381043	5381115	tRNA-Ala
54	SRO_t54	+	5519029	5519101	tRNA-Asn
55	SRO_t55	+	5519108	5519180	tRNA-Asn
56	SRO_t56	+	5519357	5519433	tRNA-Met
57	SRO_t57	+	5604508	5604582	tRNA-Val
58	SRO_t58	–	5998716	5998642	tRNA-Leu
59	SRO_t59	–	6342850	6342763	tRNA-Leu
60	SRO_t60	–	6490851	6490780	tRNA-Val
61	SRO_t61	–	6490973	6490902	tRNA-Val
62	SRO_t62	–	6491048	6490975	tRNA-Cys
63	SRO_t63	–	6491159	6491084	tRNA-Gly
64	SRO_t64	+	6497885	6497959	tRNA-Val
65	SRO_t65	+	6498710	6498784	tRNA-Val
66	SRO_t66	+	7005118	7005191	tRNA-Met
67	SRO_t67	–	7486065	7485974	tRNA-SeC(p)

Table S4. List of TTA codon-containing ORFs in the *S. rochei* chromosome.

SRO locus [a]	note	Coding region	Putative function
SRO_0031		28575..28964	TATA-binding protein associated factor Taf2
SRO_0056		54516..54824	Protein-L-isoaspartate O-methyltransferase 2
SRO_0065		62715..65615	Tetratricopeptide TPR_1 repeat-containing protein
SRO_0068		66968..67576	ATP-dependint DNA ligase
SRO_0087		complement(80551..81438)	Uncharacterized protein
SRO_0093		complement(85415..85978)	IS transposase
SRO_0096		86675..87073	IS1647-like transposase
SRO_0101		90575..90925	Uncharacterized protein
SRO_0106		93120..93563	IS1647-like transposase
SRO_0113		98942..99430	Uncharacterized protein
SRO_0148		123685..124008	Putative transposase
SRO_0156		131060..134476	Non-ribosomal peptide synthetase/polyketide synthase domain-containing protein
SRO_0159		complement(144169..144543)	Uncharacterized protein
SRO_0160		144974..145678	Peptide synthetase
SRO_0161		145675..146946	Amine oxidase
SRO_0162		146950..147747	Imidazole glycerol phosphate synthase subunit HisF
SRO_0164		148813..151926	Amino acid adenylation domain-containing protein
SRO_0167		153886..157014	Thioester reductase domain protein
SRO_0197		177519..177884	TnpA family transposase
SRO_0202		182219..183118	phytanoyl-CoA dioxygenase
SRO_0208		complement(187685..189526)	Radical SAM domain protein
SRO_0210		190859..191668	Uncharacterized protein
SRO_0211		191665..192450	Putative membrane protein
SRO_0243		218132..218743	Uncharacterized protein
SRO_0246		223271..223645	Uncharacterized protein
SRO_0252		227014..227313	Putative uncharacterized protein
SRO_0264		complement(235863..237299)	Putative uncharacterized protein
SRO_0272		complement(243816..245051)	transposase IS4 family protein
SRO_0325		299825..300424	predicted protein
SRO_0326		300546..300998	IS transposase
SRO_0337		complement(315477..316799)	Glutamate-1-semialdehyde aminotransferase
SRO_0338		complement(316964..318388)	Tryptophan 2,3-dioxygenase
SRO_0396		complement(383036..384076)	response regulator receiver protein
SRO_0397		384376..385749	l-amino acid ligase
SRO_0409		396388..397188	Uncharacterized protein
SRO_0414		400822..403530	Putative regulatory protein AfsR
SRO_0424		414050..415345	antibiotic transport protein
SRO_0447		435097..435747	RNA polymerase sigma-70 factor, sigma-E family
SRO_0494		complement(480603..480830)	Uncharacterized protein
SRO_0550		complement(565618..567453)	glycoside hydrolase family protein
SRO_0650		686010..688838	secreted protein
SRO_0707		complement(753212..754153)	myo-inositol 1-monophosphatase
SRO_0732		775656..776525	SARP family transcriptional regulator
SRO_0738		786598..788064	TMC biosynthetic enzyme R4
SRO_0768		complement(813595..814479)	Uncharacterized protein
SRO_0800		complement(846636..847130)	Uncharacterized protein
SRO_0807		complement(851457..851726)	Uncharacterized protein
SRO_0819		complement(863748..865973)	serine/threonine protein kinase
SRO_0820		866347..867090	Uncharacterized protein
SRO_0875		complement(942433..943428)	LacI-family transcriptional regulator
SRO_0937		complement(1020560..1026541)	Putative DEAD/DEAH box helicase
SRO_0940		complement(1029169..1033725)	Uncharacterized protein
SRO_0942		1034680..1037622	Type III restriction enzyme, res subunit
SRO_0945		1042188..1043366	Uncharacterized protein
SRO_0990		1088539..1088982	IS1647-like transposase
SRO_0999		complement(1100356..1101135)	branched-chain amino acid ABC transporter permease protein

SRO_1047	1163380..1164456	Uncharacterized protein
SRO_1077	complement(1196222..1196614)	Uncharacterized protein
SRO_1107	1231842..1232918	two-component system response regulator
SRO_1143	1266964..1268187	Putative uncharacterized protein
SRO_1150	1273894..1275378	Uncharacterized protein
SRO_1151	1275652..1279617	NB-ARC domain-containing protein
SRO_1152	complement(1279687..1281348)	Uncharacterized protein
SRO_1189	complement(1323183..1324307)	Uncharacterized protein
SRO_1313	complement(1454019..1455086)	Putative uncharacterized protein
SRO_1319	1463250..1465532	Non-ribosomal peptide synthetase
SRO_1321	1468741..1469994	major facilitator MFS1 protein
SRO_1422	complement(1588884..1589468)	adenylosuccinate lyase
SRO_1441	complement(1609353..1609736)	Uncharacterized protein
SRO_1443	complement(1610569..1611759)	ATP-binding region ATPase domain-containing protein
SRO_1494	complement(1676363..1676680)	Diguanylate cyclase (Precursor)
SRO_1520	complement(1701947..1702231)	CagE, TrbE, VirB component of type IV transporter system, conserved region
SRO_1545	complement(1730267..1730584)	Putative uncharacterized protein
SRO_1564	1764539..1767568	AfsR-like transcriptional regulator
SRO_1581	complement(1781850..1782716)	transcriptional regulator
SRO_1651	complement(1867888..1869678)	Uncharacterized protein
SRO_1739	complement(1970660..1971304)	A-factor receptor-like protein
SRO_1761	complement(1989927..1990805)	ABC transporter ATP-binding protein
SRO_1780	complement(2012655..2014691)	1-deoxy-D-xylulose-5-phosphate synthase
SRO_1874	complement(2122499..2122999)	Uncharacterized protein
SRO_1876	complement(2123498..2124673)	bldA-regulated nucleotide binding protein
SRO_1893	complement(2149122..2153210)	Uncharacterized protein
SRO_1894	complement(2153215..2154624)	Putative uncharacterized protein
SRO_1911	complement(2169819..2172329)	LPXTG-motif cell wall anchor domain-containing protein
SRO_1912	2172419..2173327	Uncharacterized protein
SRO_1914	2175047..2175997	iron-dependent peroxidase
SRO_1948	2207053..2209872	helicase
SRO_2013	complement(2280696..2282915)	ABC transporter
SRO_2024	complement(2292635..2294083)	drug resistance efflux protein
SRO_2050	complement(2326138..2326770)	Putative uncharacterized protein Sb08g019850
SRO_2278	2589550..2589864	Putative 2-keto-4-pentenoate hydratase/2-oxohepta-3-ene-1,7-dioic acid hydratase
SRO_2334	complement(2655136..2657472)	phosphodiesterase
SRO_2489	complement(2821956..2822219)	NERD domain-containing protein
SRO_2546	2882699..2886346	2-oxoglutarate decarboxylase
SRO_2714	3064322..3065104	integral membrane protein
SRO_2726	3079076..3079483	Uncharacterized protein
SRO_2746	3102859..3104952	magnesium or manganese-dependent protein phosphatase
SRO_2775	complement(3132173..3132970)	Hypothetical protein
SRO_2934	3298711..3300228	serine/threonine protein kinase
SRO_3063	3430240..3430905	crp/FNR family transcriptional regulator
SRO_3163	complement(3534658..3537678)	transcriptional regulator, SARP family protein
SRO_3189	3558233..3560317	Uncharacterized protein
SRO_3203	complement(3574121..3576106)	Uncharacterized protein
SRO_3204	complement(3576103..3577281)	hydrolase
SRO_3230	3597167..3598360	secreted serine-rich protein
SRO_3249	3618058..3619149	secreted cellulose binding protein
SRO_3253	3621067..3621993	xre family toxin-antitoxin system, antitoxincomponent
SRO_3300	complement(3676721..3677020)	Acriflavin resistance protein
SRO_3302	3677837..3678439	Uncharacterized protein
SRO_3343	3721791..3722267	regulator
SRO_3354	complement(3734747..3735931)	secreted hydrolase

SRO_3364	3746077..3747000	Transcriptional regulator
SRO_3379	3762114..3763352	ISXo8 transposase
SRO_3381	complement(3764448..3765092)	transcriptional regulator
SRO_3398	complement(3788305..3789174)	integral membrane protein
SRO_3498	complement(3898105..3899181)	glycosyl transferase family protein
SRO_3520	complement(3922922..3923947)	membrane transport protein
SRO_3532	3935166..3935630	Putative uncharacterized protein
SRO_3534	complement(3939138..3940505)	LigA protein
SRO_3539	3943563..3946601	Uncharacterized protein
SRO_3540	complement(3946929..3947657)	Uncharacterized protein
SRO_3600	4019739..4020731	phosphotransferase enzyme family protein
SRO_3631	4053056..4054105	Putative LuxR family regulator
SRO_3635	4055677..4056495	Uncharacterized protein
SRO_3778	4210861..4211277	integral membrane protein
SRO_3815	4247762..4248793	Uncharacterized protein
SRO_3838	4265139..4265552	Uncharacterized protein
SRO_3840	complement(4266363..4267880)	Putative restriction endonuclease
SRO_3842	complement(4269596..4271776)	Regulatory protein MerR
SRO_3843	complement(4271871..4272068)	Uncharacterized protein
SRO_3850	complement(4279320..4280165)	regulatory protein
SRO_3851	4280399..4281652	Uncharacterized protein
SRO_3934	4388838..4389038	predicted protein
SRO_3966	complement(4412655..4413497)	DNA-binding protein
SRO_4002	complement(4443156..4444259)	methylase of polypeptide chain release factors
SRO_4120	complement(4556184..4556771)	predicted protein
SRO_4163	4603167..4603583	Serine phosphatase
SRO_4180	complement(4619818..4622037)	pimeloyl-CoA synthetase
SRO_4233	4683282..4684931	PAS sensor protein
SRO_4238	complement(4689382..4690725)	tetratricopeptide TPR-2
SRO_4264	4712621..4712935	predicted protein
SRO_4280	complement(4727752..4728498)	Hypothetical protein
SRO_4398	complement(4856196..4857233)	XRE family transcriptional regulator
SRO_4406	complement(4859535..4860188)	Putative Carbonic anhydrase
SRO_4414	complement(4864644..4865519)	Uncharacterized protein
SRO_4510	complement(4982812..4985061)	glycosyl transferase
SRO_4545	5031562..5032836	Uncharacterized protein
SRO_4546	5032985..5034454	transmembrane transport protein
SRO_4585	complement(5069614..5071320)	integral membrane protein
SRO_4602	complement(5085565..5086752)	O-methyltransferase
SRO_4616	complement(5094415..5095371)	Putatibe restriction endonuclease
SRO_4703	complement(5185607..5186050)	ATPase/histidine kinase/DNA gyrase B/HSP90 domain protein
SRO_4735	complement(5218914..5219807)	Uncharacterized protein
SRO_4811	5301900..5302631	Crp/Fnr family transcriptional regulator
SRO_4817	complement(5310279..5311301)	D-isomer specific 2-hydroxyacid dehydrogenase
SRO_4855	complement(5354055..5354399)	Dyp-type peroxidase family (Precursor)
SRO_4961	complement(5465769..5467709)	amylo-alpha-1,6-glucosidase
SRO_4968	5476131..5477231	predicted protein
SRO_4970	complement(5478207..5478440)	Ankyrin repeat domain-containing protein 13C (Fragment)
SRO_4975	complement(5485702..5486019)	IS transposase
SRO_4982	5489969..5491333	plasmid transfer protein
SRO_4990	5494555..5495928	integrase
SRO_4992	5496844..5497710	NERD domain-containing protein
SRO_5018	complement(5530176..5532065)	Uncharacterized protein
SRO_5027	5542510..5546037	Putative fatty-acid-CoA ligase FADD9
SRO_5039	5557310..5558470	beta-lactamase class C
SRO_5155	5673250..5674470	Major facilitator superfamily MFS_1
SRO_5210	complement(5723604..5724620)	AraC-family transcriptional regulator
SRO_5232	5741605..5742666	Uncharacterized protein
SRO_5452	complement(5981738..5982589)	coenzyme A transferase

SRO_5453		complement(5982590..5983342)	enoyl-CoA hydratase
SRO_5496		complement(6031213..6032124)	regulatory protein
SRO_5513		complement(6049963..6050292)	Putative uncharacterized protein
SRO_5526		complement(6063244..6064467)	LacI-family transcriptional regulator
SRO_5548		6088987..6089733	nucleotidyltransferase domain-containing protein
SRO_5728		6275740..6277503	FMNH2-utilizing oxygenase
SRO_5730		complement(6298975..6299349)	Uncharacterized protein
SRO_5752		6300917..6301303	Hypothetical protein
SRO_5869		6425080..6426210	two-component system sensor kinase
SRO_5875		6432881..6433417	NUDIX hydrolase
SRO_5876		complement(6433444..6433704)	Uncharacterized protein
SRO_5918		6474454..6475170	phosphatase
SRO_5961		complement(6519332..6519565)	Uncharacterized protein
SRO_6015		6580732..6582162	amino acid permease
SRO_6047		6615254..6616210	Uncharacterized protein
SRO_6166		6745230..6752147	beta-ketoacyl synthase
SRO_6224		complement(6811730..6812593)	DNA-binding protein
SRO_6316		complement(6948174..6949532)	Albicidin efflux pump transmembrane protein
SRO_6344		complement(6980312..6981268)	Helix-turn-helix motif
SRO_6394		complement(7037379..7038863)	amino acid permease related protein
SRO_6449		complement(7100665..7101600)	integral membrane protein
SRO_6451		7102800..7104236	2-polyprenyl-6-methoxyphenol 4-hydroxylase
SRO_6585		7231755..7232720	secreted protein
SRO_6665		complement(7347121..7348578)	magnesium or manganese-dependent protein phosphatase
SRO_6793		7478656..7479900	Membrane protein
SRO_6877		complement(7562998..7563945)	peptidase M48 Ste24p
SRO_6926		complement(7612191..7612562)	Tat pathway signal sequence domain protein
SRO_6927		complement(7612690..7613886)	Uncharacterized protein
SRO_6946		7632381..7633031	tetracycline repressor
SRO_6947		7633107..7633274	Uncharacterized protein
SRO_6999		complement(7684079..7684819)	beta-glucanase/beta-glucan synthetase
SRO_7003		complement(7688884..7690467)	peptidase
SRO_7017		complement(7706054..7707058)	Uncharacterized protein
SRO_7038		complement(7728285..7729007)	transcriptional regulatory protein degU
SRO_7094		complement(7775663..7777999)	Serine/threonine protein kinase
SRO_7097		complement(7780980..7781756)	undecaprenyl pyrophosphate synthetase
SRO_7121		7811892..7812257	acetyltransferase
SRO_7146		7836104..7837561	cytochrome P450
SRO_7147		7837677..7839254	Uncharacterized protein
SRO_7191		complement(7883722..7885692)	ABC transporter ATP-binding protein
SRO_7209		7902460..7903668	cytochrome P450
SRO_7210		7903771..7905123	alpha/beta hydrolase MppK
SRO_7211		7905650..7924645	non-ribosomal peptide synthase
SRO_7215		7946023..7947273	Major facilitator transporter
SRO_7223	PemA1	7953542..7976965	polyketide synthase
SRO_7224	PemA2	7977037..7987833	polyketide synthase
SRO_7227	PemA5	8011881..8021942	modular polyketide synthase
SRO_7229	PemC	8023302..8024501	cytochrome P450 hydroxylase
SRO_7232	PemR	complement(8026047..8029616)	DnrI/RedD/AfsR family transcriptional regulator
SRO_7360		complement(8170735..8170920)	ABC transporter related protein
SRO_7366		complement(8176948..8177943)	Thymidylate synthase
SRO_7408		8219243..8219968	Uncharacterized protein
SRO_7481		complement(8283208..8283432)	AraC family transcriptional regulator
SRO_7511		complement(8307432..8307665)	Lipopolysaccharide biosynthesis protein
SRO_7538		complement(8335839..8336228)	TATA-binding protein associated factor Taf2

[a] Yellow highlights indicate TTA codon-containing ORFs possibly involved in secondary metabolite biosynthesis.

Table S5. TTA-containing ORFs in the *S. rochei* chromosome classified by their occurrence in other *Streptomyces* species.

Occurrence in *Streptomyces*	SRO number	Total
Present only in *S. rochei* 7434AN4	0031, 0056, 0065, 0068, 0087, 0101, 0113, 0148, 0156, 0159, 0160, 0161, 0162, 0164, 0167, 0197, 0202, 0208, 0210, 0211, 0243, 0246, 0252, 0264, 0272, 0337, 0338, 0396, 0397, 0409, 0414, 0424, 0447, 0494, 0550, 0650, 0732, 0738, 0768, 0800, 0807, 0819, 0820, 0875, 0937, 0940, 0942, 0945, 0999, 1047, 1077, 1107, 1143, 1150, 1151, 1152, 1189, 1313, 1319, 1321, 1441, 1443, 1494, 1520, 1545, 1564, 1761, 1874, 1876, 1893, 1894, 1911, 1912, 1948, 2013, 2024, 2050, 2278, 2489, 2714, 2726, 2746, 2775, 2934, 3063, 3163, 3189, 3253, 3300, 3302, 3354, 3364, 3379, 3381, 3498, 3520, 3532, 3534, 3539, 3540, 3600, 3631, 3635, 3778, 3815, 3838, 3840, 3842, 3843, 3850, 3934, 3966, 4120, 4163, 4233, 4238, 4280, 4398, 4406, 4414, 4545, 4546, 4585, 4602, 4616, 4703, 4735, 4811, 4817, 4855, 4968, 4970, 4990, 4992, 5018, 5027, 5039, 5155, 5210, 5232, 5452, 5453, 5496, 5513, 5526, 5548, 5728, 5730, 5875, 5918, 6166, 6316, 6344, 6394, 6449, 6451, 6585, 6793, 6877, 6926, 6927, 6946, 6947, 6999, 7003, 7017, 7038, 7094, 7097, 7121, 7146, 7147, 7191, 7215, 7224, 7229, 7360, 7366, 7408, 7481, 7511, 7538	182
Shared with *S. hygroscopicus* only	0096, 0326, 1422, 1651, 1739, 1914, 3203, 3204, 3230, 4002, 4961, 4982, 5876, 6665, 7209, 7210, 7211, 7232.	18
Shared with *S. avermitilis* only	4510, 6047, 7223, 7227	4
Shared with *S. griseus* only	3249, 3851, 4180	3
Shared with *S. scabiei* only	3343, 3398, 4264	3
Shared with *S. hygroscopicus*, and *S. avermitilis*	0106, 0990	2
Shared with *S. hygroscopicus*, and *S. scabiei*	0325, 4975	2
Shared with *S. hygroscopicus*, and *S. coelicolor*	5961	1
Shared with *S. avermitilis*, and *S. scabiei*	0707, 6015	2
Shared with *S. griseus*, and *S. scabiei*	5869	1
Shared with *S. hygroscopicus*, *S. avermitilis*, and *S. coelicolor*	93	1
Shared with *S. hygroscopicus*, *S. avermitilis*, and *S. scabiei*	2546	1
Shared with *S. hygroscopicus*, *S. coelicolor*, and *S. scabiei*	1780, 6224	2
Shared with *S. hygroscopicus*, *S. avermitilis*, *S. coelicolor*, and *S. scabiei*	2334, 5752	2
Common in all five strains	1581	1

Table S6. Protein families in *S. rochei* 7434AN4 and other four completely sequenced *Streptomyces.*

Family	SRO	SHJG	SCO	SAV	SGR
RNA polymerase ECF sigma factor	21 (0.28)	41 (0.46)	46 (0.59)	42 (0.55)	31 (0.43)
RNA polymerase sigma factor	36 (0.48)	13 (0.15)	14 (0.18)	13 (0.17)	13 (0.18)
Two-component response regulator	95 (1.26)	72 (0.81)	84 (1.07)	72 (0.95)	74 (1.04)
Two-component histidine kinase (1)	113 (1.49)	27 (0.31)	44 (0.56)	28 (0.37)	31 (0.43)
Two-component histidine kinase (2)		20 (0.23)	23 (0.29)	25 (0.33)	23 (0.32)
Serine/threonine protein kinase	62 (0.82)	31 (0.35)	26 (0.33)	26 (0.34)	24 (0.34)
Mg or Mn-dependent protein phosphatase	50 (0.66)	54 (0.61)	41 (0.52)	41 (0.54)	35 (0.49)
TetR-family transcriptional regulator	110 (1.45)	131 (1.48)	133 (1.70)	104 (1.37)	97 (1.36)
MarR-family transcriptional regulator	48 (0.63)	47 (0.53)	41 (0.52)	34 (0.45)	33 (0.46)
GntR-family transcriptional regulator	41 (0.54)	38 (0.43)	41 (0.52)	35 (0.46)	28 (0.39)
LacI-family transcriptional regulator	18 (0.24)	29 (0.33)	36 (0.46)	28 (0.37)	23 (0.32)
LysR-family transcriptional regulator	39 (0.52)	37 (0.42)	40 (0.51)	29 (0.38)	25 (0.35)
AraC-family transcriptional regulator	34 (0.45)	35 (0.40)	26 (0.33)	24 (0.32)	22 (0.31)
ROK-family transcriptional regulator	16 (0.21)	22 (0.25)	27 (0.35)	22 (0.29)	16 (0.22)
IclR-family transcriptional regulator	9 (0.12)	16 (0.18)	16 (0.20)	11 (0.15)	14 (0.20)
AsnC-family transcriptional regulator	16 (0.21)	12 (0.14)	13 (0.17)	13 (0.17)	10 (0.14)
DNA-binding protein	52 (0.69)	16 (0.18)	24 (0.31)	26 (0.34)	19 (0.27)
ABC transporter ATP-binding protein	153 (2.02)	114 (1.29)	133 (1.70)	134 (1.77)	162 (2.27)
ABC transporter permease protein	156 (2.06)	72 (0.81)	83 (1.06)	74 (0.98)	53 (0.74)
ABC transporter sugar-binding protein	29 (0.38)	16 (0.18)	16 (0.20)	14 (0.18)	13 (0.18)
MFS-family transporter	152 (2.01)	132 (1.49)	99 (1.27)	88 (1.16)	97 (1.36)
Short chain dehydrogenase	79 (1.04)	103 (1.16)	81 (1.04)	73 (0.96)	57 (0.80)
Alpha/beta hydrolase	76 (1.00)	46 (0.52)	35 (0.45)	38 (0.50)	28 (0.39)
Cytochrome P450	42 (0.55)	34 (0.38)	18 (0.23)	32 (0.42)	27 (0.38)
Zn-dependent dehydrogenase	44 (0.58)	59 (0.67)	37 (0.47)	44 (0.58)	27 (0.38)
Aldehyde dehydrogenase	25 (0.33)	30 (0.34)	21 (0.27)	23 (0.30)	22 (0.31)
Acyl-CoA synthetase	46 (0.61)	46 (0.52)	25 (0.32)	40 (0.53)	37 (0.52)
Acyl-CoA dehydrogenase	38 (0.50)	17 (0.19)	18 (0.23)	25 (0.33)	27 (0.38)
Luciferase family monooxygenase	30 (0.40)	33 (0.37)	14 (0.18)	26 (0.34)	20 (0.28)
FAD-dependent monooxygenase	24 (0.32)	24 (0.27)	22 (0.28)	13 (0.17)	12 (0.17)
Aldo/keto reductase	29 (0.38)	29 (0.33)	25 (0.32)	21 (0.28)	19 (0.27)
Methyltransferase	135 (1.78)	23 (0.26)	21 (0.27)	24 (0.32)	27 (0.38)
Enoyl-CoA hydratase	18 (0.24)	13 (0.15)	8 (0.10)	16 (0.21)	14 (0.20)
Aminotransferase	61 (0.81)	17 (0.19)	16 (0.20)	14 (0.18)	14 (0.20)

Numbers indicate annotated protein families on the basis of BLASTP search. Parenthesis indicates percentage of families in their genome. Data for four reference strains was cited from Ref. 19 [Wu, *et al*., *BMC Genomics* **13**, 337 (2012)]. SRO, *S. rochei* 7434AN4; SHJG, *S. hygroscopicus* 5008; SCO, *S. coelicolor* A3(2); SAV, *S. avermitilis* MA-4680; SGR, *S. griseus* IFO13350.

Table S7. Location of CRISPR-associate (*cas*) genes and CRISPR repeats in the *S. rochei* chromosome.

	Locus tag	Positions	Functions
CRISPR repeats #1		2,205,703 ... 2,206,837	CRISPR repeat sequences
cas genes	SRO_1948	2,207,053 ... 2,209,872	CRISPR-associated helicase/endonuclease Cas3
	SRO_1949	2,210,208 ... 2,211,800	CRISPR-associated protein, Cse1 family
	SRO_1950	2,211,867 ... 2,212,598	Type I-E CRISPR-associated protein Cse2/CasB
	SRO_1951	2,212,668 ... 2,213,861	Type I-E CRISPR-associated protein Cas7/Cse4/CasC
	SRO_1952	2,213,858 ... 2,214,733	Type I-E CRISPR-associated protein Cas5/CasD
	SRO_1953	2,214,730 ... 2,215,422	Type I-E CRISPR-associated protein Cas6/Cse3/CasE
	SRO_1954	2,215,419 ... 2,216,408	Type I-E CRISPR-associated endonuclease Cas1
	SRO_1955	2,216,433 ... 2,216,750	Type I-E CRISPR-associated endoribonuclease Cas2
CRISPR repeats #2		2,216,850 ... 2,217,182	CRISPR repeat sequences

Table S8. Sequences of CRISPR repeats in the *S. rochei* chromosome.

No.	Start position	5'-3' Sequences (DR Consensus sequence: CGGTTCACCTCCGCCTGCGCGGAGCGGAC)
		CRISPR repeat #1 (nt 2,205,703 – 2,206,837)
1	2,205,703	AATCGCACCTCCGCCTGCGCAGAGCGGACTCCAGGTCGGCCCGGTGCACCCC
2	2,205,755	CGGCTCACCTCCGCCTGCGCGGAGCGGACACGACTGGACCGAGCCGCCCTGGTTCTGCTCGCAGATCT
3	2,205,823	CGGTTCACCTCCGCCTGCGCGGAGCGGACTCCAGCTCTTCGCGGGCGGTCTCGCGTACCTC
4	2,205,884	CGGTTCACCTCCGCCTGCGCGGAGCGGACTCACCCCCGCGACTGACCAGGTGGCCTCGT
5	2,205,943	GGCGGTTCACCCGCCTGCGCGGAGCGGACCCGTCCGGGAGCTGCGTCGCCAGCACCAGCGC
6	2,206,004	CGGTTCACCTCCGCCTGCGCGGAGCGGACAGCTCGGGCGACATGAGCGGGTACTCGGCATC
7	2,206,065	CGGTTCACCTCCGCCTGCGCGGAGCGGACGGGGTGCCGGTGCCGGTGCCGGTGACGTTGGCCTTGGCCGCGTT
8	2,206,138	CGGTTCACCTCCGCCTGCGCGGAGCGGACGTCATCACCCGATTCTCCCGGTGCAGGGGGCT
9	2,206,199	CGGTTCACCTCCGCCTGCGCGGAGCGGACGCCGGCGACGAGGGCGGCGTCGACCATGGCTG
10	2,206,260	CGGTTCACCTCCGCCTGCGCGGAGCGGACGCGATGCGCAGCTTCTCGGCCTCCTCGGCAGC
11	2,206,321	CGGTTCACCTCCGCCTGCGCGGAGCGGACTGCGAAGTTCACAATGACCCCGGTCGCAGAGT
12	2,206,382	CGGTTCACCTCCGCCTGCGCGGAGCGGACGTCTTCACGAACACCGGTACCGCCATACCCAC
13	2,206,443	CGGTTCACCTCCGCCTGCGCGGAGCGGACCGCGCCTGCCTGCTCGCCGTCGAGTCCGCCGA
14	2,206,504	CGGTTCACCTCCGCCTGCGCGGAGCGGACGCCTTCTTGCCGGTGGCCCTGTACTCCAGGGC
15	2,206,565	CGGTTCACCTCCGCCTGCGCGGAGCGGACGTCCCCGCTCCCCGCTCCCCGAGAGGCCCCCA
16	2,206,626	TGGTTCACCTCCGCCTGCGCGGAGCGGACAGTGTCGGTGTGCTGGTAGGAGGTGGAGAGGA
17	2,206,687	CGGTTCACCTCCGCCTGCGCGGAGCGGACGCCCGCACACCGCCCACCCGCAGCACGGCCCG
18	2,206,748	CGGTTCACCTCCGCCTGCGCGGAGCGGACCGTAGGACGCTCCGCAGGAGCTCCTCGCCGGT
19	2,206,809	CGGTTCACCTCCGCCTGCGCGGAGCGGAC
		CRISPR repeat #2 (nt 2,216,850 – 2,217,182)
20	2,216,850	GGGTTCACCTCCGCCTGCGCGGAGCGGACACGCGGAAGCTGCTCTTCGTGACCGCGCTGGA
21	2,216,911	CGGTTCACCTCCGCCTGCGCGGAGCGGACGACGGAATCACCCGAAGGTTCTCGGGCCATC
22	2,216,971	CGGTTCACCTCCGCCTGCGCGGAGCGGACTACGGCGGTGTCGGTGCAGGCGGGCCTGCCGT
23	2,217,032	CGGTTCACCTCCGCCTGCGCGGAGCGGACGCCCGTGCTCCACCACTCGGCATGTCGGTCTC
24	2,217,093	CGGTTCACCTCCGCCTGCGCGGAGCGGACGTCGCCCGTGGGCGGCGGCCCGGAATGGACAA
25	2,217,154	CGGTTCACCTCCGCCTGCGCGGAGCGGAC
